# High-Yield Production of 4-Hydroxybenzoate From Glucose or Glycerol by an Engineered *Pseudomonas taiwanensis* VLB120

**DOI:** 10.3389/fbioe.2019.00130

**Published:** 2019-06-12

**Authors:** Christoph Lenzen, Benedikt Wynands, Maike Otto, Johanna Bolzenius, Philip Mennicken, Lars M. Blank, Nick Wierckx

**Affiliations:** ^1^Institute of Applied Microbiology iAMB, RWTH Aachen University, Aachen, Germany; ^2^Forschungszentrum Jülich, Institute of Bio- and Geosciences IBG-1: Biotechnology, Jülich, Germany

**Keywords:** 4-hydroxybenzoate, aromatics, metabolic engineering, *Pseudomonas taiwanensis* VLB120, shikimate pathway, glycerol, synthetic biology

## Abstract

Aromatic compounds such as 4-hydroxybenzoic acid are broadly applied in industry for a myriad of applications used in everyday life. However, their industrial production currently relies heavily on fossil resources and involves environmentally unfriendly production conditions, thus creating the need for more sustainable biotechnological alternatives. In this study, synthetic biology was applied to metabolically engineer *Pseudomonas taiwanensis* VLB120 to produce 4-hydroxybenzoate from glucose, xylose, or glycerol as sole carbon sources. Genes encoding a 4-hydroxybenzoate production pathway were integrated into the host genome and the flux toward the central precursor tyrosine was enhanced by overexpressing genes encoding key enzymes of the shikimate pathway. The flux toward tryptophan biosynthesis was decreased by introducing a P290S point mutation in the *trpE* gene, and degradation pathways for 4-hydroxybenzoate, 4-hydroxyphenylpyruvate and 3-dehydroshikimate were knocked out. The resulting production strains were tailored for the utilization of glucose and glycerol through the rational modification of central carbon metabolism. In batch cultivations with a completely mineral medium, the best strain produced 1.37 mM 4-hydroxybenzoate from xylose with a C-mol yield of 8% and 3.3 mM from glucose with a C-mol yield of 19.0%. Using glycerol as a sole carbon source, the C-mol yield increased to 29.6%. To our knowledge, this is the highest yield achieved by any species in a fully mineral medium. In all, the efficient conversion of bio-based substrates into 4-hydroxybenzoate by these deeply engineered *P. taiwanensis* strains brings the renewable production of aromatics one step closer.

## Introduction

Aromatic compounds are broadly applied in every-day life. Being the basic substance of the paraben group, 4-hydroxybenzoate and its derivatives mainly serve as a preservative in cosmetic as well pharmaceutical products, and beyond that, it is used for the manufacturing of Vectran fibers (Beers and Ramirez, 1990; Menczel et al., 1997). Currently, 4-hydroxybenzoate is produced commercially via the Kolbe-Schmitt-reaction from potassium phenoxide and carbon dioxide (Lindsey and Jeskey, 1957). This way of production, however, harbors major drawbacks in that fossil resources are used as substrates and that the reaction takes place under harsh conditions. Due to relatively low and isomerically poor product yields (Lindsey and Jeskey, 1957), improved production strategies using toxic ionic liquids have been developed (Zhao et al., 2007; Benaskar et al., 2009; Stark et al., 2009). In total, these routes render the overall process non-sustainable and the production conditions hazardous and environmentally unfavorable. Biotechnological production of 4-hydroxybenzoate using microbial cell factories, however, would overcome these issues, since renewable substrates such as glucose, xylose, or glycerol are converted under far milder conditions. Nature offers a variety of candidates for the production of aromatics and other bulk and fine chemicals. Among those, Pseudomonads recently have gained great biotechnological interest (Sun et al., 2006; Elbahloul and Steinbüchel, 2009; Loeschcke and Thies, 2015; Tiso et al., 2016; Nikel and de Lorenzo, 2018). In comparison to other bacteria, these Gram-negative soil bacteria exhibit a high tolerance toward organic solvents (Kieboom et al., 1998), and due to their versatile metabolism, they accept a great variety of substrates (Dos Santos et al., 2004; Wierckx et al., 2015). Furthermore, sophisticated tools for genetic manipulation are available (Martínez-García and de Lorenzo, 2011; Silva-Rocha et al., 2013; Martínez-García et al., 2014; Nikel et al., 2014b; Belda et al., 2016) and cultivation conditions are undemanding. Different *Pseudomonas* strains have been used for the production of a range of bio-based aromatics including anthranilate, phenylalanine and derivatives, 4-coumarate, phenazines, 4-hydroxystyrene, and phenol (Wierckx et al., 2005; Nijkamp et al., 2007; Verhoef et al., 2009; Kuepper et al., 2015; Schmitz et al., 2015; Molina-Santiago et al., 2016; Wynands et al., 2018).

In microbes, 4-hydroxybenzoate can be produced in two ways. On the one hand, chorismate, which is formed via the shikimate pathway, can be converted into 4-hydroxybenzoate and pyruvate by the chorismate-pyruvate-lyase (EC 4.1.3.40, UbiC). This reaction was already exploited in *Escherichia coli, Corynebacterium glutamicum, Klebsiella pneumoniae*, and *Pseudomonas putida* KT2440 (Müller et al., 1995; Barker and Frost, 2001; Yu et al., 2016; Kallscheuer and Marienhagen, 2018; Kitade et al., 2018; Syukur Purwanto et al., 2018). However, in order to assure sufficient flux toward product formation, shutting down competing chorismate consuming pathways is often required. This often results in auxotrophic production strains which require co-feeding of aromatic amino acids or complex medium components such as yeast extract. By overexpression of an *ubiC* gene from *E. coli*, Müller et al. (1995) produced 0.12 g l^−1^ 4-hydroxybenzoate from 18 g l^−1^ glucose with a strain of *K. pneumoniae* which was auxotrophic for all aromatic amino acids. Likewise, Barker and Frost (2001) developed an *E. coli* production strain deficient in biosynthesis of tyrosine, phenylalanine and tryptophan and were able to accumulate 4-hydroxybenzoate with a C-mol yield of 15.2% during fed-batch fermentation. A *P. putida* KT2440 biocatalyst engineered by Yu et al. (2016) achieved a 4-hydroxybenzoate titer of 1.7 g l^−1^ corresponding to a C-mol yield of 18.1%. However, this strain lacked the ability to natively produce tryptophan as well as phenylpyruvate, so that, as in the case for the aforementioned hosts, additional supplementation of intermediates was necessary. Recently, Kallscheuer and Marienhagen (2018) metabolically engineered a *C. glutamicum* strain and produced 3.3 g l^−1^ 4-hydroxybenzoate in shake flasks, which corresponds to a C-mol yield of 12.6%. Using the same species as a platform, Kitade et al. (2018) conducted growth-arrested fermentations by which a 4-hydroxybenzoate titer of 36.6 g l^−1^ and a C-mol yield of 47.8% could be reached. Remarkably, this was achieved without the introduction of auxotrophies by employing a novel UbiC variant from *Providencia rustigianii* which is insensitive to product inhibition. However, this very high yield was achieved in a 2-step process where biomass was first grown on a rich medium, followed by 4-hydroxybenzoate production in a mineral medium using a high density (5% wet weight) of biomass from the rich medium culture. The 1st-stage culture in rich medium, and thus all biomass formation, was not accounted for in the yield calculation. Syukur Purwanto et al. (2018) produced 137.6 mM 4-hydroxybenzoate with a C-mol yield of 9.65% during fed-batch cultivations using an engineered *C. glutamicum* strain. Due to the deletion of the *trpE* gene, however, supplementation of tryptophan was required, and high 4-hydroxybenzoate titers were not reached in a fully mineral medium.

On the other hand, 4-hydroxybenzoate biosynthesis can also be accomplished via tyrosine. The first step of this pathway is deamination of tyrosine into 4-coumarate through a tyrosine ammonia-lyase (EC 4.3.1.23, TAL). Subsequently, 4-coumarate is further processed by moonlight activities of feruloyl-CoA synthetase (EC 6.2.1.34, Fcs), enoyl-CoA hydratase (EC 4.2.1.17, Ech), and vanillin dehydrogenase (EC 1.2.1.67, Vdh) to yield 4-hydroxybenzoate (Harwood and Parales, 1996; Jiménez et al., 2002). Although this approach has a lower maximum theoretical yield due to the loss of one C-atom as CO_2_ in the conversion of chorismate to tyrosine, it has the benefit of not requiring auxotrophies since tyrosine is the endpoint of the metabolic pathway rather than a central intermediate as in the case of chorismate. Also, chorismate was shown to be non-enzymatically rearranged to phenylpyruvate in engineered phenylalanine/tyrosine auxotrophic *Saccharomyces cerevisiae* strains, thus lowering its availability as a substrate for production (Winter et al., 2014). Further, both chorismate and tyrosine are key precursors for the production of many other aromatics, thus making it useful to have platform strains that efficiently channel carbon flux toward both of these metabolites. Following the route via tyrosine, Verhoef et al. (2007) accomplished 4-hydroxybenzoate formation with a C-mol yield of 19.3% during shake flask cultivations using glycerol as a sole carbon source and 11.0% with glucose with a modified strain of *P. putida* S12, whereas fed-batch cultivations using glycerol as a sole carbon source lead to a C-mol yield of 8.5%. Further improvement was achieved through the deletion of the *hpd* gene coding for the 4-hydroxyphenylpyruvate dioxygenase, resulting in a C-mol yield of 13.4% on glucose (Verhoef et al., 2010). Implementation of a xylose-degrading pathway and subsequent laboratory evolution resulted in a strain that was able to form 4-hydroxybenzoate with C-mol yield of 12.4% on xylose, 17.5% on glucose, and 19.3% on glycerol in shake flask experiments, whereas cultivation in fed-batch mode using a mixed-substrate feeding strategy with glycerol and xylose achieved a C-mol yield of 16.3% 4-hydroxybenzoate (Meijnen et al., 2011).

**Figure 1:**
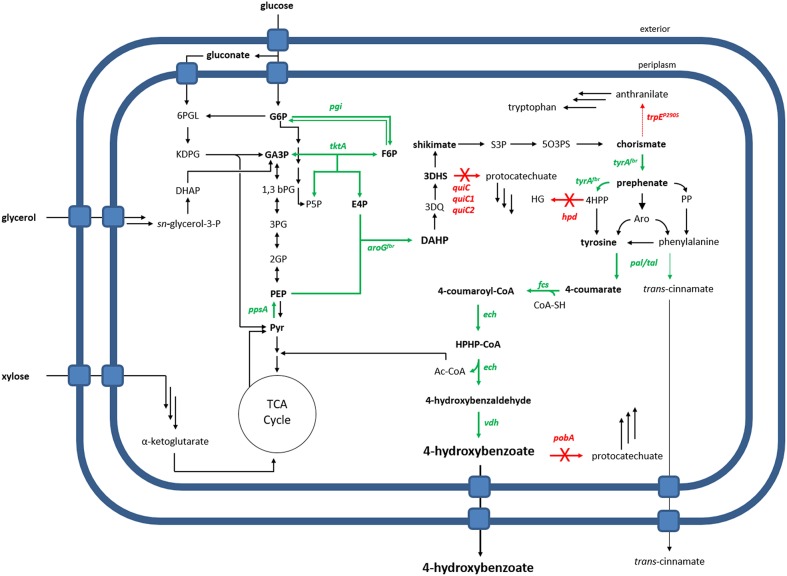
Metabolic overview of 4-hydroxybenzoate production by an engineered *P. taiwanensis* VLB120. Overexpressed genes are shown as green arrows, deleted genes as red arrows with a cross. The dashed red arrow symbolizes a point mutation resulting in a flux decrease. 6PGL, 6-phosphogluconate; G6P, glucose-6-phosphate; KDPG, 2-keto-3-deoxy-6-phosphogluconate; GA3P, glyceraldehyde-3-phosphate; F6P, fructose-6-phosphate; S7P, sedoheptulose-7-phosphate; E4P, erythrose-4-phosphate; P5P, pentose-5-phosphate; 1,3bPG, 1,3-bisphosphoglycerate; 3PG, 3-phosphoglycerate; 2PG, 2-phosphoglycerate; PEP, phosphoenolpyruvate; Pyr, pyruvate; DAHP, 3-Deoxy-D-arabinoheptulosonate 7-phosphate; 3DQ, 3-dehydroquinate; 3DHS, 3-dehydroshikimate; S3P, shikimate-3-phosphate; 5O3PS, 5-O-(1-carboxyvinyl)-3-phosphoshikimate; 4HPP, 4-hydroxyphenylpyruvate; PP, phenylpyruvate; Aro, arogenate; HG, homogentisate; HPHP-CoA, 4-Hydroxyphenyl-β-hydroxypropionyl-CoA; Genes: *pgi*, glucose-6-phosphate isomerase; *ppsA*, phosphoenolpyruvate synthase; *tktA*, transketolase; *aroG*, DAHP synthase; *tyrA*, prephenate dehydrogenase; *quiC*, 3-dehydroshikimate dehydratase; *trpE*, anthranilate synthase; *hpd*, 4-hydroxyphenylpyruvate dioxygenase; *tal*, tyrosine ammonia-lyase; *pal*, phenylalanine ammonia-lyase; *fcs*, feruloyl-CoA synthetase; *ech*, enoyl-CoA dehydratase; *vdh*, vanillin dehydrogenase; *pobA, p*-hydroxybenzoic acid hydroxylase.

In this study, *Pseudomonas taiwanensis* VLB120 was subject to metabolic engineering in order to generate a whole-cell biocatalyst for the production of 4-hydroxybenzoate via tyrosine (Figure 1). This species is naturally capable of using xylose as a sole carbon source (Köhler et al., 2015), rendering it a superior candidate for conversion of lignocellulosic feedstock compared to other Pseudomonads. For the generation of an efficient 4-hydroxybenzoate production host, we downregulated and disrupted competing pathways, and we overexpressed key precursor-supplying genes through stable genomic integration. Primary metabolic genes were also overexpressed, tailored for the use of glucose or glycerol as sole carbon source, and the intrinsic use of xylose as a substrate for 4-hydroxybenzoate production was demonstrated.

## Materials and Methods

### Chemicals

Unless indicated otherwise, all chemicals were purchased from Sigma Aldrich (Taufkirchen, Germany) or Carl Roth (Karlsruhe, Germany).

### Bacterial Strains and Culture Conditions

All strains used in this work are listed in Table 1. *E. coli* strains were cultivated in Lysogeny Broth (LB) medium (10 g l^−1^ N-Z-Amine, 5 g l^−1^ yeast extract, 5 g l^−1^ NaCl). When grown on plates, agar was added to a final concentration of 1.5% before autoclaving. When selection was necessary, the medium was supplemented with the respective antibiotics: 50 mg l^−1^ kanamycin, 10 mg l^−1^ gentamicin, 100 mg l^−1^ ampicillin, 10 mg l^−1^ tetracycline. Gentamicin concentration was raised to 30 mg l^−1^ during selection on agar plates. Shake flasks were filled with 10% of the flask volume and incubated at 37°C and 300 rpm. All *Pseudomonas* strains were grown on LB plates or cetrimide agar plates and selected with the respective antibiotics as described for *E. coli*, at a temperature of 30°C.

**Table 1:** Strains used or engineered in this study.

**Strain**	**Characteristics**	**References**
*E. coli* PIR1	F^−^Δ*lac169 rpoS(Am) robA1 creC510 hsdR514 endA recA1uidA(ΔMluI)::pir-116*; host for oriV(R6K) vectors in high copy number	Thermo Fisher Scientific
*E. coli* DH5α λpir	λpir lysogeny of DH5α	Glover and Hames, 1985
*E. coli* DH5α pTNS1	*supE44 ΔlacU169 (φ80 lacZΔM15) hsdR17 (rk- mk+) recA1 endA1 gyrA96 thi-1 relA1*	de Lorenzo lab
*E. coli* HB101 pRK2013	Helper strain carrying plasmid pRK2013; F^−^λ^−^*hsdS20(rB^−^ mB^−^) recA13 leuB6(Am) araC14 Δ(gpt-proA)62 lacY1 galK2(Oc) xyl-5 mtl-1 thiE1 rpsL20(SmR) glnX44(AS)*	Figurski and Helinski, 1979
*E. coli* DH5α pBBFLP	Strain used for excision of kanamycin resistance cassette F^−^*endA1 glnV44 thi-1 recA1 relA1 gyrA96 deoR nupG purB20* φ80d*lacZ*ΔM15 Δ(*lacZYA-argF*)U169, hsdR17(*r_*K*_*^−^*m_*K*_*^+^), λ^−^	de las Heras et al., 2008
*P. taiwanensis* VLB120	Wild type	Panke et al., 1998
*P. taiwanensis* VLB120 CL1	Derived from *P. taiwanensis* VLB120; Deficient for *pobA, hpd*; carrying genes *ech, vdh* and *fcs* randomly integrated via Tn5 transposon.	This study
*P. taiwanensis* VLB120 CL1 *Rt*PAL	*P. taiwanensis* VLB120 CL1 carrying plasmid pJT'*Rt*PAL	This study
*P. taiwanensis* VLB120 CL1 *Fj*TAL	*P. taiwanensis* VLB120 CL1 carrying plasmid pJT'*Fj*TAL	This study
*P. taiwanensis* VLB120 CL1 *Tc*XAL	*P. taiwanensis* VLB120 CL1 carrying plasmid pJT'*Tc*XAL	This study
*P. taiwanensis* VLB120 CL1 *Rs*TAL	*P. taiwanensis* VLB120 CL1 carrying plasmid pJT'*Rs*TAL	This study
*P. taiwanensis* VLB120 CL1.1 *Rt*PAL	Derived from *P. taiwanensis* VLB120 CL1; Deficient for *quiC, quiC1, quiC2*; carrying plasmid pJT'*Rt*PAL	This study
*P. taiwanensis* VLB120 CL2	Derived from *P. taiwanensis* VLB120 CL1.1; Point mutation in *trpE* resulting in P290S substitution	This study
*P. taiwanensis* VLB120 CL2 *Rt*PAL	*P. taiwanensis* VLB120 CL2 carrying plasmid pJT'*Rt*PAL	This study
*P. taiwanensis* VLB120 CL2 *Rt*PALa	*P. taiwanensis* VLB120 CL2 carrying plasmid pJT'*Rt*PALa	This study
*P. taiwanensis* VLB120 CL2 *Rt*PALat	*P. taiwanensis* VLB120 CL2 carrying plasmid pJT'*Rt*PALat	This study
*P. taiwanensis* VLB120 CL2 *Rt*PALatt	*P. taiwanensis* VLB120 CL2 carrying plasmid pJT'*Rt*PALatt	This study
*P. taiwanensis* VLB120 CL2 BG14a *Rt*PALat	*P. taiwanensis* VLB120 CL2 carrying genes *RtPAL, aroG^*fbr*^, tyrA^*fbr*^* at *attTn7* site under control of 14a promoter	This study
*P. taiwanensis* VLB120 CL2 BG14d *Rt*PALat	*P. taiwanensis* VLB120 CL2 carrying genes *RtPAL, aroG^*fbr*^, tyrA^*fbr*^* at *attTn7* site under control of 14d promoter	This study
*P. taiwanensis* VLB120 CL2 BG14e *Rt*PALat	*P. taiwanensis* VLB120 CL2 carrying genes *RtPAL, aroG^*fbr*^, tyrA^*fbr*^* at *attTn7* site under control of 14e promoter	This study
*P. taiwanensis* VLB120 CL3	*P. taiwanensis* VLB120 CL2 carrying genes *RtPAL, aroG^*fbr*^, tyrA^*fbr*^* at *attTn7* site under control of 14g promoter	This study
*P. taiwanensis* VLB120 CL2 BG14fg *Rt*PALat	*P. taiwanensis* VLB120 CL2 carrying genes *RtPAL, aroG^*fbr*^, tyrA^*fbr*^* at *attTn7* site under control of 14fg promoter	This study
*P. taiwanensis* VLB120 CL2 BG14ffg *Rt*PALat	*P. taiwanensis* VLB120 CL2 carrying genes *RtPAL, aroG^*fbr*^, tyrA^*fbr*^* at *attTn7* site under control of 14ffg promoter	This study
*P. taiwanensis* VLB120 CL3.1	*P. taiwanensis* VLB120 CL3 carrying plasmid pBNT'*ppsA*	This study
*P. taiwanensis* VLB120 CL3.2	*P. taiwanensis* VLB120 CL3 carrying plasmid pBNT'*pgi*	This study
*P. taiwanensis* VLB120 CL3.3	*P. taiwanensis* VLB120 CL3 carrying plasmid pBNT'*ppsA-pgi*	This study
*P. taiwanensis* VLB120 CL4	Derived from *P. taiwanensis* VLB120 CL4; Carrying genes *RsTAL, aroG^*fbr*^, tyrA^*fbr*^* at *attTn7* site under control of 14f promoter	This study
*P. taiwanensis* VLB120 CL4.3	*P. taiwanensis* VLB120 CL4 carrying plasmid pBNT'*ppsA-pgi*	This study

For production experiments in batch mode, two liquid precultures were prepared. At first, 500 ml flasks containing 50 ml LB medium were inoculated with cells from a freshly grown LB agar plate or glycerol stock and incubated overnight at 30°C and 300 rpm. The second preculture was conducted in 500 ml flasks containing 50 ml mineral salts medium (MSM) (Hartmans et al., 1989) (buffer: 11.64 g l^−1^ K_2_HPO_4_, 4.89 g l^−1^ NaH_2_PO_4_. Nitrogen source: 2 g l^−1^ (NH_4_)_2_SO_4_. Trace elements: 10 mg l^−1^ EDTA, 100 mg l^−1^ MgCl_2_ · 6 H_2_O, 2 mg l^−1^ ZnSO_4_ · 7 H_2_O, 1 mg l^−1^ CaCl_2_ · 2 H_2_O, 5 mg l^−1^ FeSO_4_ · 7 H_2_O, 0.2 mg l^−1^ Na_2_MoO_4_ · 2 H_2_O, 0.2 mg l^−1^ CuSO_4_ · 5 H_2_O, 0.4 mg l^−1^ CoCl_2_ · 6 H_2_O, 1 mg l^−1^, MnCl_2_ · 2 H_2_O. The medium contained either 20 mM glucose, 40 mM glycerol, or 24 mM xylose as sole carbon source, unless stated otherwise. When using glycerol as a sole carbon source in the main culture, the preculture contained 40 mM glycerol and 5 mM glucose to decrease the lag phase on glycerol. All MSM precultures were incubated for 16–20 h at 300 rpm and 30°C and inoculated into the main culture to a final OD_600_ of 0.1. For the main culture, cells from the MSM preculture were harvested via centrifugation at 5,000 rpm and 4°C for 10 min and washed twice with 0.9% NaCl. Subsequently, 24-well System Duetz plates (Duetz et al., 2000) containing 1.5 ml fresh MSM per well and 0.2 mM IPTG for induction of expression of the ferulic genes (*ech, vdh*, and *fcs*) and, if necessary, 0.1 mM salicylate for induction of pBNT plasmids, were inoculated to a final OD_600_ of 0.1 and incubated at 30°C and 300 rpm. Unless indicated otherwise, all strains were cultivated in biological triplicates, and the standard error of the mean was used for indication of error bars. Pulsed fed-batch cultivations using MSM with glycerol as a sole carbon source were conducted with a Biostat® A Plus system (Sartorius Stedim, Göttingen, Germany) with a maximum volume of 2 l. Initial batch phase took place in 1 l MSM containing 120 mM glycerol and twice the amount of mineral salts. Initial stirrer speed was set to 480 rpm and aeration to 1 vvm. Dissolved oxygen tension was regulated at >30% through a stirrer cascade and continuously monitored using an InPro 6800 amperometric oxygen sensor (Mettler Toledo, Columbus, USA). During the course of cultivation, this value was maintained by mixing in pure oxygen using manual control. Monitoring of the pH was carried out with a 405-DPAS-SC-K8S/225 pH electrode (Mettler Toledo, Columbus, USA) and maintained at pH 7 by titration of 4 M HCl and 2 M NH_4_OH. During fed-batch phase, glycerol was pulsed through a syringe to a final concentration of 200 mM each time dissolved oxygen tension increased and glycerol in the fermenter was consumed.

### DNA Techniques

All primers (see Table S1) were designed either with Clone Manager Professional (Sci-Ed, Denver, USA) or NEBuilder® Assembly Tool (New England Biolabs, Ipswich, USA) and purchased from eurofins Genomics (Ebersberg, Germany). Codon-optimization for genes *RsTAL* (*Rhodobacter sphaeroides* ATCC 17025), *FjTAL* (*Flavobacterium johnsoniae* ATCC 17061), and *TcXAL* (*Trichosporon cutaneum* ATCC 90039) for *P. taiwanensis* VLB120 was carried out using the OPTIMIZER online tool (Puigbò et al., 2007), whereas preferences were set as follows: genetic code: eubacterial; method: guided random; manual exclusion of undesired restriction sites and rare codons exhibiting a usage of <6%. Optimized DNA fragments were ordered and purchased from Thermo Fisher Scientific (Thermo Fisher Scientific, Waltham, USA) or Integrated DNA Technologies (Coralville, USA). Inserts for all plasmids were amplified via PCR using Q5® High-Fidelity DNA polymerase (New England Biolabs, Ipswich, USA). Likewise, this was done for backbone plasmids, when cloning took place using the NEBuilder® HiFi DNA Assembly Master Mix (New England Biolabs, Ipswich, USA). When vectors were assembled using T4 DNA Ligase (Thermo Fisher Scientific, Waltham, USA), backbone DNA was isolated from an *E. coli* overnight culture using Monarch® Plasmid Miniprep Kit (New England Biolabs, Ipswich, USA). Subsequently, all DNA fragments were digested with the respective restriction enzymes (New England Biolabs, Ipswich, USA) and purified with the Monarch® PCR & DNA Cleanup Kit (New England Biolabs, Ipswich, USA). Assembled and purified plasmids (Table 2) were transferred into *E. coli* and *Pseudomonas* via transformation, whereas conjugation was used for *Pseudomonas* as well. For *E. coli*, heat-shock transformation was done according to a protocol by Sambrook et al. (1989) Conjugation was performed through triparental mating using a streamlined method as outlined by Wynands et al. (2018). Selection of *Pseudomonas* took place on cetrimide agar plates. When pEMG and pBELK plasmids were transferred, helper strain *E. coli* HB101 pRK2013 was used, whereas this strain as well as DH5α pTNS1 were taken for conjugation of pBG14-based constructs.

**Table 2:** Plasmids used and constructed in this work.

**Plasmid**	**Characteristics**	**References**
pJT'mcs	Amp^R^ Gm^R^, expression vector, under control of P_tac_ promoter	Nijkamp et al., 2007
pTacPAL	Amp^R^ Gm^R^, expression vector, harboring *Rt*PAL under control of P_tac_ promoter	Nijkamp et al., 2007
pBELK	Mini-Tn5 delivery vector; tnpA oriV(R6Kγ) oriT(RK2) lacI^Q^ P_trc_ bla F*RT*-aphA-F*RT*, Amp^R^ Km^R^	Nikel and de Lorenzo, 2013
pBELK ferulic s	Mini-Tn5 delivery vector harboring genes *ech, vdh* and *fcs* from *P. putida* S12	This study
pJT'*Rs*TAL	pJT expression vector harboring *RsTAL*	This study
pJT'*Fj*TAL	pJT expression vector harboring *FjTAL*	This study
pJT'*Tc*XAL	pJT expression vector harboring *TcXAL*	This study
pJT *Rt*PAL	pJT expression vector harboring *RtPAL*	This study
pBWatt	pSEVA234 expression vector harboring genes *aroG^*fbr*^, tyrA^*fbr*^, tktA*; Km^R^, ori pBBR1, *laqI^*q*^-P_*trc*_*	This study
pJT'*Rt*PALa	pJT expression vector harboring gene *aroG^*fbr*^*	This study
pJT'*Rt*PALat	pJT expression vector harboring gene *aroG^*fbr*^, tyrA^*fbr*^*	This study
pJT'*Rt*PALatt	pJT expression vector harboring gene *aroG^*fbr*^, tyrA^*fbr*^, tktA*	This study
pBG14a	Tn7 delivery vector; KmR GmR, ori *R6K, Tn7L*, and *Tn7R* flanks, BCD2–*msfgfp* fusion, promoter no. 28	Zobel et al., 2015
pBG14d	Tn7 delivery vector; KmR GmR, ori *R6K, Tn7L*, and *Tn7R* flanks, BCD2–*msfgfp* fusion, promoter no. 51	Zobel et al., 2015
pBG14e	Tn7 delivery vector; KmR GmR, ori *R6K, Tn7L*, and *Tn7R* flanks, BCD2–*msfgfp* fusion, promoter no. 17	Zobel et al., 2015
pBG14f	Tn7 delivery vector; KmR GmR, ori *R6K, Tn7L*, and *Tn7R* flanks, BCD2–*msfgfp* fusion, promoter no. 25	Zobel et al., 2015
pBG14g	Tn7 delivery vector; KmR GmR, ori *R6K, Tn7L*, and *Tn7R* flanks, BCD2–*msfgfp* fusion, promoter no. 42	Zobel et al., 2015
pBG14fg	Tn7 delivery vector; KmR GmR, ori *R6K, Tn7L*, and *Tn7R* flanks, BCD2–*msfgfp* fusion, hybrid promoter composed of No. 25 & No. 42	Köbbing et al. in preparation
pBG14ffg	Tn7 delivery vector; KmR GmR, ori *R6K, Tn7L*, and *Tn7R* flanks, BCD2–*msfgfp* fusion, hybrid promoter 2 x No. 25 & No. 42	Köbbing et al. in preparation
pBG14a *Rt*PALat	Tn7 delivery vector harboring genes *RtPAL, aroG^*fbr*^, tyrA^*fbr*^*, promoter no. 28	This study
pBG14d *Rt*PALat	Tn7 delivery vector harboring genes *RtPAL, aroG^*fbr*^, tyrA^*fbr*^*, promoter no. 51	This study
pBG14e *Rt*PALat	Tn7 delivery vector harboring genes *RtPAL, aroG^*fbr*^, tyrA^*fbr*^*, promoter no. 17	This study
pBG14g *Rt*PALat	Tn7 delivery vector harboring genes *RtPAL, aroG^*fbr*^, tyrA^*fbr*^*, promoter no. 42	This study
pBG14fg *Rt*PALat	Tn7 delivery vector harboring genes *RtPAL, aroG^*fbr*^, tyrA^*fbr*^*, hybrid promoter no. 25 & no. 42	This study
pBG14ffg *Rt*PALat	Tn7 delivery vector harboring genes *RtPAL, aroG^*fbr*^, tyrA^*fbr*^*, hybrid promoter 2 × no. 25 & no. 42	This study
pBG14f *Rs*TALat	Tn7 delivery vector harboring genes *RsTAL, aroG^*fbr*^, tyrA^*fbr*^*, promoter no. 25	This study
pRK2013	Helper plasmid for conjugational transfer; Km^R^, *oriV*(RK2/ColE1), *mob^+^ tra^+^*	Figurski and Helinski, 1979
pTNS1	Helper plasmid; Amp^R^, *ori* R6K, *TnSABC+D* operon	Choi et al., 2005
pSW-2	Gm^R^, *ori*RK2, *xylS, Pm* → I-*sce*I (transcriptional fusion of I-*sce*I to *Pm*)	Martínez-García and de Lorenzo, 2011
pBBFLP	Helper plasmid for excision of antibiotic markers, *oriV*(pBBR1), *oriT*(RK2) RK2 *mob^+^* λ*P_*R*_*::*FLP* λ(*cI*857) *sacB tet*, Tet^R^	de las Heras et al., 2008
pEMG *pobA*	Knockout vector carrying TS1 and TS2 flanking regions of *p*-hydroxybenzoic acid hydroxylase (*pobA*)	Wynands et al., 2018
pEMG *hpd*	Knockout vector carrying TS1 and TS2 flanking regions of 4-hydroxyphenylpyruvate dioxygenase (*hpd*)	Wynands et al., 2018
pEMG *quiC*	Knockout vector carrying TS1 and TS2 flanking regions of dehydroshikimate dehydratase (*quiC*)	Wynands et al., 2018
pEMG *quiC1*	Knockout vector carrying TS1 and TS2 flanking regions of dehydroshikimate dehydratase (*quiC1*)	Wynands et al., 2018
pEMGu *quiC2*	Knockout vector carrying TS1 and TS2 flanking regions dehydroshikimate dehydratase (*quiC2*)	Wynands et al., 2018
pEMGu *trpE*^P290S^	Knockout vector carrying TS1 and TS2 flanking regions of the codon for the P290S substitution of *trpE* gene	Wynands et al., 2018
pBNT'mcs	Expression vector harboring salicylate-inducible nagR/P_NagAa_ promoter, Km^R^	Verhoef et al., 2010
pBNT'*ppsA*	pBNT expression vector harboring gene encoding phosphoenolpyruvate synthase (*ppsA*)	This study
pBNT'*pgi*	pBNT expression vector harboring gene encoding glucose-6-phosphate isomerase (*pgi*)	This study
pBNT'*ppsA-pgi*	pBNT expression vector harboring gene encoding phosphoenolpyruvate synthase (*ppsA*), and glucose-6-phosphate isomerase (*pgi*)	This study

Gene knockouts were carried out according to a protocol developed by Martínez-García and de Lorenzo (2011). The pEMG suicide vectors containing TS1 and TS2 flanking regions of the gene to be eliminated were conjugated into the respective recipient strain, whereas successful integration was verified via colony PCR. Due to high ampicillin resistance of *P. taiwanensis* VLB120, plasmid pSW-2 encoding enzyme I-SceI was used instead of pSW-I. Induction of expression by addition of 3-methylbenzoate was not required. Knockouts were confirmed by colony PCR and DNA sequencing. Genes *ech, vdh*, and *fcs*, were amplified from the genome of *P. putida* S12 (Hartmans et al., 1989) and randomly integrated into the genome of *P. taiwanensis* VLB120 via the pBELK Tn5 mini-transposon system developed by Nikel and de Lorenzo (2013). Integration sites were identified through arbitrary-primed PCR as described by Martínez-García et al. (2014) using the Q5® DNA polymerase and subsequent DNA sequencing and BLAST analysis against the genome of *P. taiwanensis* VLB120. In order to excise the kanamycin resistance cassette, plasmid pBBFLP harboring a flippase gene was transformed into selected clones and loss of resistance was confirmed via selection of Km^s^ clones on LB agar plates. Stable integration of key genes and modulated expression was achieved by using the Tn7-based calibrated promoter system developed by Zobel et al. (2015).

Cloning procedures were routinely verified via colony PCR using *OneTaq*® DNA polymerase (New England Biolabs, Ipswich, USA). Colonies were picked from transformation plates and lysed in 30 μl PEG200 (pH 12) for 5–10 min (Chomczynski and Rymaszewski, 2006).

### Analytical Methods

Optical densities of cell cultures were measured at a wavelength of 600 nm using an Ultrospec 10 spectrophotometer (GE Healthcare, Chicago, USA). Cell dry weight was determined by multiplying OD_600_ values by the empirical factor 0.505. Aromatics were analyzed by HPLC. Samples taken during cultivations were centrifuged at 17,000 *g* for 2 min and the supernatant was filtered using syringe filters with a pore size of 0.2 μm. After addition of methanol (Th. Geyer, Renningen, Germany) in a 1:1 ratio, samples were stored at 4°C overnight in order to precipitate any salts or proteins. After another centrifugation at 13,000 rpm for 2 min, the supernatant was taken for analysis. HPLC was performed using a System Gold 168 diode array detector (Beckman Coulter, Brea, USA) and an ISAspher 100-5 C18 BDS reversed phase HPLC column (ISERA, Düren, Germany) at 30°C and a flow rate of 0.8 ml min. Elution took place with a gradient starting at 95% of 0.1% (v/v) TFA and 5% methanol for 2 min, followed by gradual increase to 100% methanol over 18 min. After 2 min at 100% methanol, initial ratios were reached again within 2 min. UV detection of aromatics was conducted at a wavelength of 260 nm. Glycerol concentrations were determined using the Glycerol GK Assay Kit (Megazyme, Bray, Ireland). Glucose concentrations were determined via HPLC using an Aminex Ion Exclusion HPX-87H column (Bio-Rad, Hercules, USA) and a Smartline RI detector 2300 (Knauer, Berlin, Germany), whereas isocratic elution took place in 5 M H_2_SO_4_ at 1.2 ml/min and 70°C. For calculation of production rates, CDW was estimated by multiplying OD values with the empirical factor 0.505. The increase of 4-hydroxybenzoate concentration between two time points was then divided by average biomass of those time points and the period of time. Growth data of the 4-hydroxybenzoate pulse experiment carried out in the Growth Profiler® (Enzyscreen, Heemstede, Netherlands) were normalized by subtracting the initial offset values of each curve compared to that of the cultivation with no 4-hydroxybenzoate pulse, thereby setting all initial points to the same value.

## Results and Discussion

### Establishing 4-Hydroxybenzoate Production in *P. taiwanensis*

One important requirement for an efficient biocatalyst to be functional is that degradation of the product itself, as well as that of potential precursors, is prevented. Wierckx et al. (2008) and Verhoef et al. (2010) showed that producers of aromatic compounds had upregulated metabolic pathways for the degradation of 4-hydroxybenzoate, protocatechuate, and tyrosine. Therefore, the *pobA* gene (PVLB_11545) responsible for the conversion of 4-hydroxybenzoate into protocatechuate, and the *hpd* gene (PVLB_11760), encoding the 4-hydroxyphenylpyruvate dioxygenase, were knocked out, rendering the cells unable to use 4-hydroxybenzoate and tyrosine as a sole carbon sources (Wynands et al., 2018). In doing so, the strain *P. taiwanensis* VLB120 Δ*pobA*Δ*hpd* was constructed. Unlike many Pseudomonads, the genome of *P. taiwanensis* VLB120 does not contain genes encoding a ferulic acid degradation pathway, and indeed this strain is unable to grow on ferulate. Since this pathway is necessary to convert 4-coumarate into 4-hydroxybenzoate, the operon containing ferulic genes *fcs, ech*, and *vdh* was amplified from the genome of *P. putida* S12 and cloned into the pBELK transposon vector (Nikel and de Lorenzo, 2013) under the control of the IPTG-inducible P_trc_ promoter. Subsequently, this operon was randomly integrated into the genome of *P. taiwanensis* VLB120 Δ*pobA*Δ*hpd*. Three clones were randomly picked and cultivated in MSM containing 20 mM glucose and 3 mM 4-coumarate. Under these conditions, all three strains produced 4-hydroxybenzoate at a rate of 0.45 mmol ${\text{g}}_{\text{CDW}}^{-1}$ h^−1^, proving the functionality of the pathway encoded by the ferulic operon. Another variant of the ferulic operon containing the additional genes encoding feruloyl-CoA dehydrogenase (*fcd*) and β-ketothiolase (*aat*) was also tested, but the addition of these two genes did not affect 4-hydroxybenzoate production rates from 4-coumarate, and they were therefore omitted in further strain engineering (data not shown). The integration sites of the operon were identified by arbitrary PCR and DNA sequencing as described in Martínez-García et al. (2014). The transposons had integrated 1,049 bp downstream from the start codon of the gene encoding the plug domain of a TonB-dependent receptor (PVLB_16205, clone 1), 242 bp downstream from the start codon of a radical SAM protein (PVLB_20680, clone 2), and 1,696 bp downstream from the start codon of a gene encoding a sensory box protein (PVLB_25350, clone 3). A map depicting the integration sites can be found in Figure S1. Since no detectable differences regarding growth or production could be observed among the different clones, a negative effect of disruption of these genes on production performance can be excluded. All further experiments were carried out with clone 1, which hereafter is named *P. taiwanensis* VLB120 CL1.

**Figure 2:**
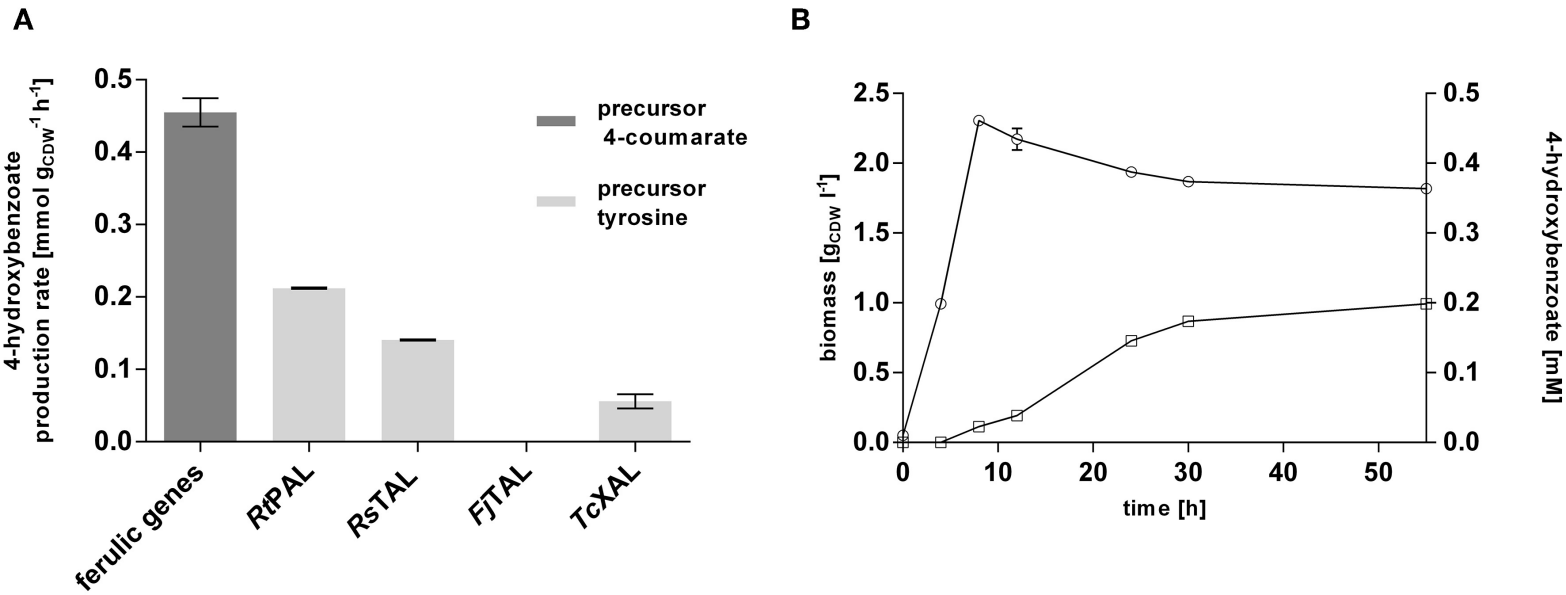
Evaluation of the 4-hydroxybenzoate production pathway in *P. taiwanensis* VLB120 CL1 in co-feeding experiments. **(A)** Comparison of the conversion rates of the two different pathway components (encoded by the ferulic operon and the ammonia-lyase genes). Cells were grown in biological triplicates in MSM with 5 mM glucose in System Duetz plates at 30°C and 300 rpm. After ~6 h, 3 mM 4-coumarate (ferulic operon) or tyrosine (PAL and TAL genes), and another 20 mM glucose were added. Highest measured rates are indicated. **(B)** 4-hydroxybenzoate production (squares) and growth (circles) of *P. taiwanensis* VLB120 CL1 (containing *Rt*PAL). Cells were cultivated in triplicates in MSM supplemented with 20 mM glucose as a sole carbon source at 30°C and 300 rpm in System Duetz plates. Expression of ferulic genes was induced with 0.2 mM IPTG. Error bars indicate standard error of the mean.

This strain was subsequently equipped with different ammonia-lyases which catalyze the conversion of tyrosine into 4-coumarate, including a native *pal* from *Rhodosporidium toruloides* (*Rt*PAL; Verhoef et al., 2007) and codon-optimized versions of a *tal* from *Rhodobacter sphaeroides* (*Rs*TAL; Xue et al., 2007), from *Flavobacterium johnsoniae* (*Fj*TAL), and a *xal* from *Trichosporon cutaneum* (*Tc*XAL) (Jendresen et al., 2015), cloned into plasmid pJT'mcs (Nijkamp et al., 2007) under the control of the constitutive P_tac_ promoter. This setup of genomic integration of the ferulic operon and plasmid-based expression of PAL/TAL-encoding genes was chosen because the ammonia-lyase is generally the rate-limiting step (Verhoef et al., 2007; Jendresen et al., 2015), and expression from a multicopy plasmid was expected to increase its activity. Co-feeding experiments with 20 mM glucose and 3 mM tyrosine confirmed the ammonia-lyase reaction as the rate-limiting step of the pathway. Among the four tested lyases, *Rt*PAL enabled the highest 4-hydroxybenzoate production rate (0.20 mmol ${\text{g}}_{\text{CDW}}^{-1}$ h^−1^), reaching 44% of the conversion velocity of the downstream pathway encoded by the ferulic operon (Figure 2A). The second best performance was exhibited by the strain harboring the *Rs*TAL construct with a rate of 0.14 mmol ${\text{g}}_{\text{CDW}}^{-1}$ h^−1^. When cultivated with 20 mM glucose in mineral medium without precursor supplementation (Figure 2B), the strain harboring the ferulic operon and the *Rt*PAL construct produced 0.20 mM 4-hydroxybenzoate, corresponding to a C-mol yield of 1.2% with a maximum production rate of 0.004 mmol ${\text{g}}_{\text{CDW}}^{-1}$ h^−1^, which is only 2% of the rate achieved with additional feed of tyrosine. However, considerable accumulation of 4-hydroxybenzoate could only be detected after cells had reached stationary phase, suggesting that at least some of the pathway genes are catabolite-repressed. Indeed, the recognition sequence motif (5′-AANAANAA-3′) characteristic for catabolite-repressed expression (Moreno et al., 2009b, 2015) could be found 37 base pairs downstream from the start codon of *fcs*, encoding the first enzymatic step in 4-coumarate conversion, and 20 bp upstream from the start codon of *ech*, which is the first gene downstream from the promoter. These findings are in accordance to those described by Verhoef et al. (2010). However, the latter site was deleted during the cloning into the pBELK vector. Upon binding of the Crc global regulator protein in conjunction with the Hfq chaperone to this motif on the mRNA level, translation is prevented (Wolff et al., 1991; MacGregor et al., 1996; Hester et al., 2000a,b; Morales et al., 2004; Ruiz-Manzano et al., 2005; Moreno et al., 2009a; Hernández-Arranz et al., 2016). Knockout of *crc* or *hfq* in order to override catabolite-repression, however, did not beneficially influence 4-hydroxybenzoate production (Figure S2). In summary, an efficient 4-hydroxybenzoate production pathway from tyrosine could be implemented in *P. taiwanensis* VLB120 CL1, in which the selection of an efficient ammonia-lyase was a key enabling factor. The fact that feeding of precursors such as tyrosine results in a greatly increased 4-hydroxybenzoate production rate suggests that, as expected, the *de novo* formation of tyrosine is a bottleneck for efficient 4-hydroxybenzoate production from glucose or glycerol.

### Enhancing Supply of Tyrosine

**Figure 3:**
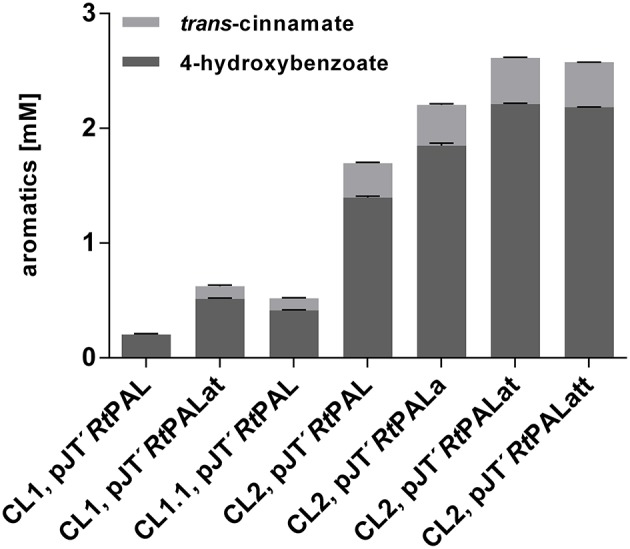
Comparison of aromatics titers of *P. taiwanensis* VLB120 CL1, *P. taiwanensis* CL1.1, and *P. taiwanensis* CL2 with overexpression of different bottleneck genes. All strains were cultivated for 96 h in biological triplicates in MSM containing 20 mM glucose as a sole carbon source in System Duetz plates at 30°C and 300 rpm, expression of ferulic genes was induced with 0.2 mM IPTG. Error bars indicate standard error of the mean.

As in the case of *pobA* and *hpd*, upregulation of 3-dehydroshikimate (3DHS) dehydratase (*quiC1*) (Peek et al., 2017) was observed in the phenol producing strain *P. putida* S12 TPL3 (Wierckx et al., 2008), converting 3DHS, one of the upper metabolites of the shikimate pathway, into protocatechuate. Recently, investigations on quinate metabolism in *P. taiwanensis* VLB120 revealed the presence of a total of three genes encoding 3DHS dehydratase (Wynands et al., 2018). Therefore, genes *quiC* (PVLB_18200)*, quiC1* (PVLB_10935), and *quiC2* (PVLB_13075) were knocked out in *P. taiwanensis* VLB120 CL1 in order to prevent the degradation of aromatic pathway intermediates, thus generating VLB120 CL1.1. Besides tyrosine, tryptophan is also formed via the shikimate pathway. In *P. putida* S12 TPL3, genome analysis revealed a mutation in the gene encoding the anthranilate synthase (*trpE*), resulting in a P290S amino acid exchange (Wierckx et al., 2008). This mutation substantially increased the carbon flux toward tyrosine in a *P. taiwanensis*-based phenol overproducer (Wynands et al., 2018). Therefore, the same point mutation as described in Wynands et al. (2018) resulting in a P290S substitution was introduced in *P. taiwanensis* VLB120 CL1.1, thereby generating *P. taiwanensis* VLB120 CL2. These genetic modifications together with the pJT'*Rt*PAL construct lead to an almost 2-fold increase in 4-hydroxybenzoate titer for *P. taiwanensis* VLB120 CL1.1 and 6.9-fold for *P. taiwanensis* VLB120 CL2 compared to *P. taiwanensis* VLB120 CL1, corresponding to C-mol yields of 2.4 and 8.1%, respectively (Figure 3). The large increase in production obtained with CL2 confirms the importance of the *trpE*^P290S^ mutation. The difference between CL1 and CL1.1 indicates that indeed a substantial portion of the carbon flux into the shikimate pathway is diverted into the 3DHS degradation pathway, thereby creating a futile cycle. These strains also produced 0.10 ± 0.01 mM (CL1.1) and 0.30 ± 0.01 mM (CL2) of *trans-*cinnamate due to loose substrate specificity of the used *Rt*PAL enzyme which is capable of converting both tyrosine and phenylalanine.

Former studies in *E. coli* revealed genes *aroG, tyrA*, and *tktA* as key steps in increasing tyrosine supply (Kikuchi et al., 1997; Li et al., 1999; Lütke-Eversloh and Stephanopoulos, 2007; Kim et al., 2014). Therefore, along with the gene encoding *Rt*PAL, feedback-resistant versions of *aroG* and *tyrA* (*aroG*^fbr^, with a D146N substitution, and *tyrA*^fbr^, with A354V and M53I substitutions) as well as *tktA*, from *E. coli*, were codon-optimized and cloned into the pJT'mcs vector. In the experiment shown in Figure 3, synergistic effects of these genes on 4-hydroxybenzoate production were investigated in MSM with 20 mM glucose as sole carbon source. When using the pJT'*Rt*PALat construct (overexpression of *Rt*PAL, *aroG*^fbr^, and *tyrA*^fbr^) in VLB120 CL1, the 4-hydroxybenzoate titer was raised to 0.51mM with a C-mol yield of 3%. Strain *P. taiwanensis* VLB120 CL2 harboring the same pJT'*Rt*PALat plasmid produced 2.21 ± 0.01 mM 4-hydroxybenzoate with a C-mol yield of 12.9%, confirming that a major contribution to enhanced production is made by downregulation and elimination of competing pathways (*quiC, quiC1, quiC2*, and *trpE*^P290S^), but also showing that the additional expression of both *aroG*^fbr^ and *tyrA*^fbr^ significantly enhances production. Compared to these strains, the inclusion of *aroG*^fbr^ alone shows an intermediate phenotype. The additional overexpression of the *tktA* gene (pJT'*Rt*PALatt) did not further increase the product concentration. The highest *trans-*cinnamate concentration of 0.40 ± 0.01 mM was found for *P. taiwanensis* VLB120 CL2 harboring pJT'*Rt*PALat, making up 15.9% of the total aromatics produced.

### Stable Genomic Integration of 4-Hydroxybenzoate Production Modules

**Figure 4:**
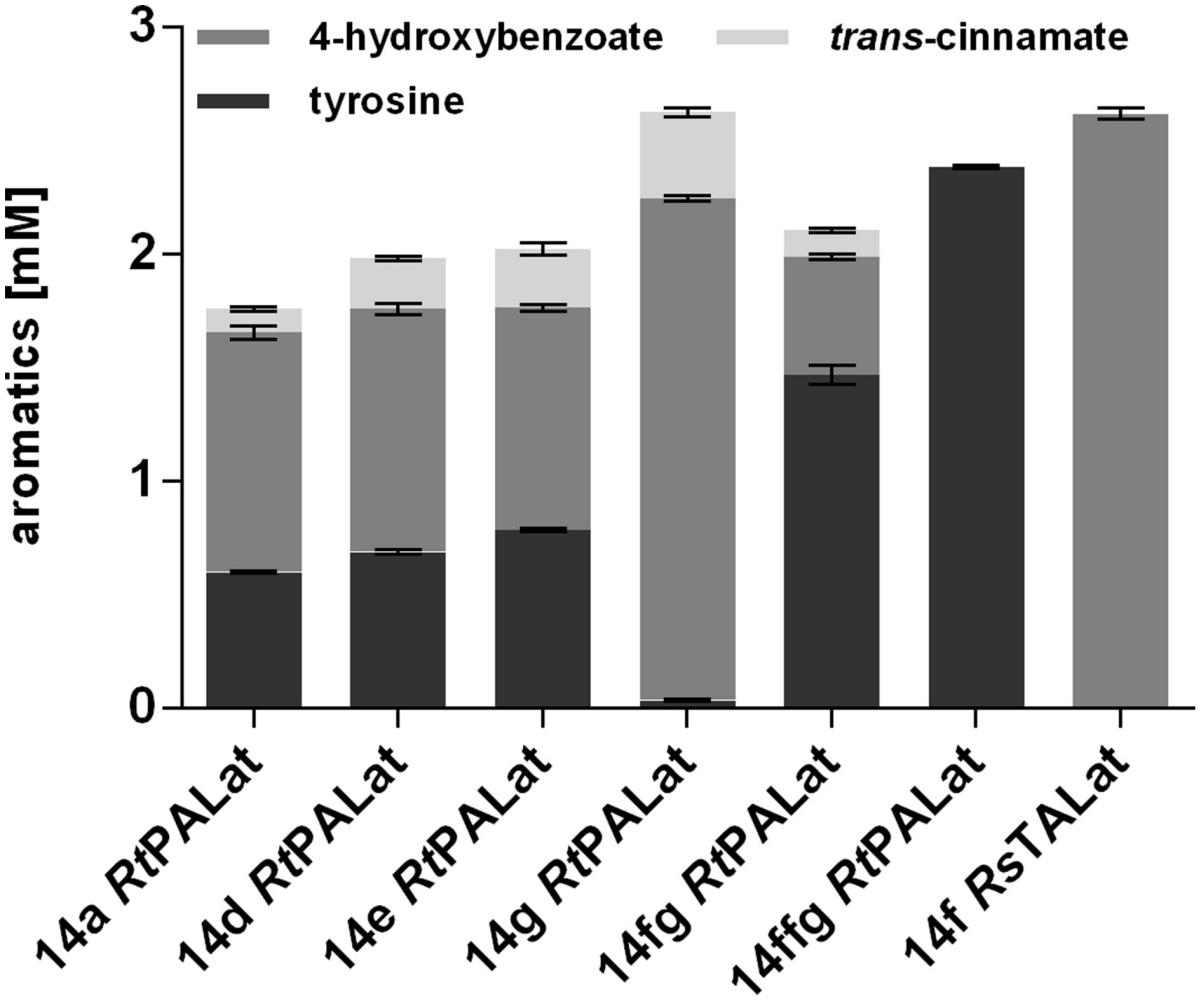
Modulation of expression of genomically integrated *RtPAL, RsTAL, aro*G^*fbr*^, and *tyr*A^*fbr*^. Increasing promoter strengths are listed from left to right. Cells were cultivated in biological triplicates in MSM containing 20 mM glucose as a sole carbon source at 30°C and 300 rpm in System Duetz plates, expression of ferulic genes was induced with 0.2 mM IPTG. Titers of final samples (96 h) are shown. Error bars indicate standard error of the mean.

The use of plasmid-based expression systems in a bioprocess has the disadvantage that selective pressure has to be maintained throughout the entire cultivation, thus leading to increased burden (Mi et al., 2016). Furthermore, plasmids inherently vary in their copy number from cell to cell, which results in greater variability and instability (Gao et al., 2014; Jahn et al., 2014). To address these issues, a Tn7 transposon system with a calibrated promoter library (Zobel et al., 2015) was used in order to stably integrate one copy of *RtPAL, aroG*^fbr^, and *tyrA*^fbr^ into the genome of *P. taiwanensis* VLB120 CL2. Different promoter strengths were tested in order to investigate optimal constitutive expression levels (Figure 4). In addition, the 14fg and 14ffg variants were included, which are stacked promoters with activities higher than 14g (Sebastian Köbbing, RWTH Aachen University, personal communication). The highest final 4-hydroxybenzoate concentration was achieved with the 14g construct, which reached a titer equal to that of the plasmid-based system. Unexpectedly, the strongest investigated promoter 14ffg resulted in a tyrosine titer of 2.4 mM, but no accumulation of 4-hydroxybenzoate or *trans-*cinnamate. Since this promoter exhibits 80% more activity than the BG14g promoter, one explanation for this may be that expression levels of *Rt*PAL were too high, leading to the selection of mutated constructs. The strain harboring the P_14g_ *Rt*PALat construct exhibited the best performance and is hereafter designated *P. taiwanensis* VLB120 CL3.

Although the *Rt*PAL enzyme enabled the highest production rate of 4-hydroxybenzoate in co-feeding experiments, one major disadvantage of this enzyme is its relaxed substrate specificity, causing it to also convert phenylalanine into *trans-*cinnamate. Thus, not only is a fraction of valuable precursor converted into a byproduct, it also hampers the downstream purification of 4-hydroxybenzoate. In an attempt to circumvent this issue, *Rt*PAL was replaced by a codon-optimized gene encoding the tyrosine ammonia-lyase from *Rhodobacter sphaeroides* (*Rs*TAL) (Xue et al., 2007). Although this TAL supports a lower 4-hydroxybenzoate production rate (Figure 2), it has a much higher relative affinity for tyrosine, thus potentially generating less byproduct. The gene was cloned along with *aroG*^fbr^ and *tyrA*^fbr^ into the pBG14f transposon vector and integrated at the *att*Tn7 site into the genome of *P. taiwanensis* VLB120 CL2, thereby generating *P. taiwanensis* VLB120 CL4. The construct containing the 14f promoter was chosen because cloning attempts using stronger promoters failed. After 96 h of cultivation in MSM containing 20 mM glucose, a 4-hydroxybenzoate titer of 2.62 ± 0.03 mM was reached, with no detectable formation of *trans-*cinnamate. The increase of 4-hydroxybenzoate corresponds to the amount of *trans-*cinnamate produced by the same strain using *Rt*PAL, indicating that in this case the *Rs*TAL enzyme efficiently redirected the flux toward the main product, likely aided by the phenylalanine hydroxylase PhhAB which converts phenylalanine into tyrosine (Arias-Barrau et al., 2004).

### Enhanced 4-Hydroxybenzoate Production Through Overexpression of *ppsA* and *pgi*

Overall, the biosynthesis of one mole of tyrosine requires one mole of erythrose-4-phosphate (E4P) and two moles of phosphoenolpyruvate (PEP). In *E. coli*, glucose is taken up through a phosphotransferase system which converts PEP into pyruvate. This system poses a severe drain on PEP, and enhanced production of tyrosine could be achieved by overexpression of the *ppsA* gene encoding phosphoenolpyruvate synthase A, which re-converts pyruvate into PEP (Yi et al., 2002; Lütke-Eversloh and Stephanopoulos, 2007; Juminaga et al., 2012). Although Pseudomonads do not take up glucose via a phosphotransferase system (Romano et al., 1970), they mainly metabolize glucose via the Entner-Doudoroff pathway (Nikel et al., 2015), which yields one PEP and one pyruvate per glucose molecule. The Embden-Meyerhof-Parnas pathway employed by *E. coli* yields two PEP per mole of glucose, and thus both organisms theoretically yield the same net production of one PEP and one pyruvate per mole of transported glucose through their major metabolic pathway. In order to increase the PEP precursor supply, we overexpressed *ppsA* in the 4-hydroxybenzoate producing strains. The gene was amplified from the genome of *P. taiwanensis* VLB120 and cloned into the pBNT'mcs expression vector (Verhoef et al., 2010) under the control of the salicylate-inducible NagR/P_nagAa_-promoter system. The second precursor E4P is formed by transketolase (*tktA*), the overexpression of which did not increase 4-hydroxybenzoate production (Figure 3). In Pseudomonads, the flux through the pentose phosphate pathway (PPP) is generally low (Fuhrer et al., 2005; Wierckx et al., 2009; Nikel et al., 2015), and enhancement of the flux through the PPP through evolution on xylose also increased 4-hydroxybenzoate production in an engineered *P. putida* S12 (Meijnen et al., 2011), making it likely that this pathway poses a bottleneck in the engineered *P. taiwanensis* strains as well. Given that overexpression of *tktA* did not improve production, phosphoglucose isomerase (*pgi*) was chosen as upstream target, converting glucose-6-phosphate into fructose-6-phosphate.

**Figure 5:**
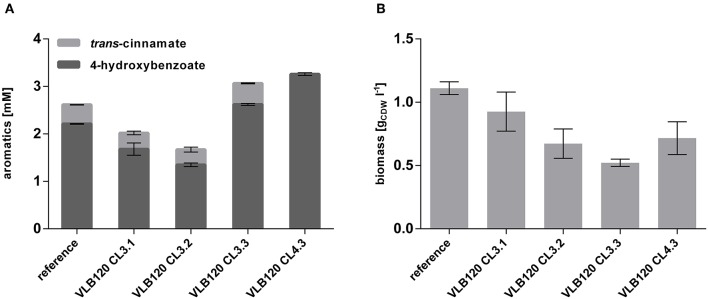
Overexpression of *ppsA* and *pgi* in *P. taiwanensis* VLB120 CL3 and CL4. Cells were cultivated in biological triplicates in MSM with 20 mM glucose as a sole carbon source at 30°C and 300 rpm in System Duetz plates. Expression of *ppsA* and *pgi* was induced with 0.1 mM salicylate and expression of the ferulic genes with 0.2 mM IPTG. Concentrations of aromatics **(A)** and biomass **(B)** measured after 96 h. Error bars indicate standard error of the mean.

To investigate whether the overexpression of these two genes has a beneficial effect on 4-hydroxybenzoate formation, they were cloned separately and together into the pBNT'mcs plasmid and transformed into *P. taiwanensis* VLB120 CL3, thereby generating *P. taiwanensis* VLB120 CL3.1 overexpressing *ppsA, P. taiwanensis* VLB120 CL3.2 overexpressing *pgi* and *P. taiwanensis* VLB120 CL3.3 overexpressing both genes. The strains were cultivated in mineral medium containing 20 mM glucose as a sole carbon source (Figure 5). Overexpression of the individual genes did not result in an increase of 4-hydroxybenzoate titer. However, a synergistic effect of both genes could be observed, by which a 4-hydroxybenzoate concentration of 2.62 ± 0.07 mM was produced from 20 mM glucose, corresponding to a C-mol yield of 15.3%, which is an increase of 18.6% compared to the equivalent strain without *ppsA-pgi* overexpression. As the best performance of 4-hydroxybenzoate production was achieved upon combined overexpression of both *ppsA* and *pgi*, the *Rs*TAL-harboring strain *P. taiwanensis* VLB120 CL4 was equipped with plasmid pBNT'*ppsA-pgi* and hereafter named *P. taiwanensis* VLB120 CL4.3. Under the same conditions as strain *P. taiwanensis* VLB120 CL3.3, this strain produced 3.26 ± 0.05 mM 4-hydroxybenzoate with a C-mol yield of 19.0%. Compared to the reference strain, overexpression of *ppsA* and/or *pgi* had a negative effect on growth. On the one hand, this may be due to the additional plasmid-borne metabolic load. However, since lowest biomass values can be observed in strains harboring *ppsA-pgi* constructs, it can be assumed that poorer growth may be due to reduced availability of PEP and E4P which is now channeled into the shikimate pathway and toward 4-hydroxybenzoate. In addition, PpsA phosphorylates pyruvate through the conversion of ATP into AMP (Berman and Cohn, 1970), and may thus constitute an ATP-wasting futile cycle in conjunction with the reverse pyruvate kinase reaction (Hädicke et al., 2015).

### Production of 4-Hydroxybenzoate Is Enhanced on Glycerol, but Not on Xylose

**Figure 6:**
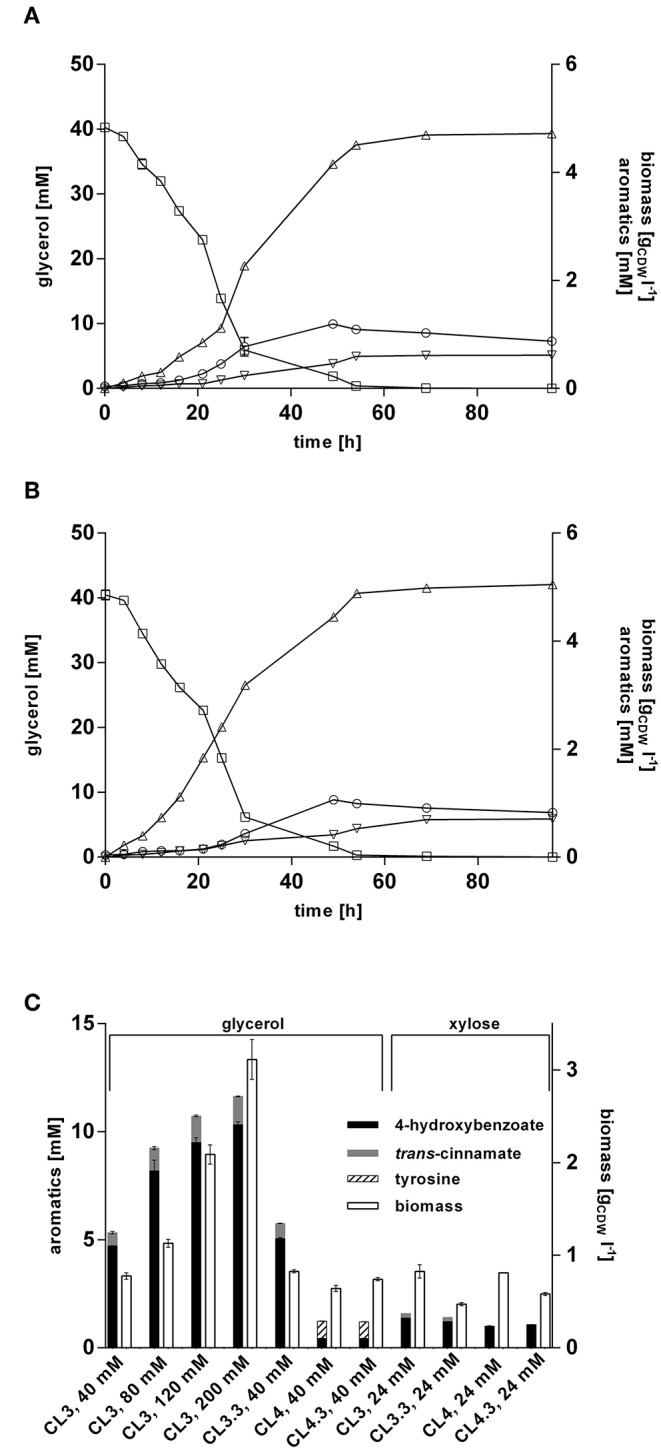
4-hydroxybenzoate production from glycerol and xylose by engineered *P. taiwanensis* VLB120 strains. Cells were grown in biological triplicates in MSM containing glycerol or 24 mM xylose as a sole carbon source in 24-well System Duetz plates at 30°C and 300 rpm. Expression of ferulic genes was induced with 0.2 mM IPTG. Cultivations of **(A)** strain CL3 and **(B)** CL3.3 Values of biomass (circles), glycerol (squares), 4-hydroxybenzoate (triangles), and *trans*-cinnamate (inverted triangles) are shown. **(C)** Aromatics concentrations and biomass after 96 h. The used substrate concentrations are indicated on the X-axis. For *P. taiwanensis* VLB120 CL3.3 and CL4.3, expression of *ppsA-pgi* was induced with 0.1 mM salicylate. Error bars indicate standard error of the mean.

Former studies showed that glycerol represents a promising alternative to glucose as a renewable biotechnological feedstock (Murarka et al., 2008; Yang et al., 2012; West, 2013; Zambanini et al., 2017; Wynands et al., 2018). Being a byproduct formed during biodiesel production, it is a cheap substrate, and another advantage over glucose is that it can be applied in higher concentrations for *Pseudomonas* batch cultivations, since no gluconate is formed which acidifies the medium. To evaluate the performance of *P. taiwanensis* VLB120 CL3 and *P. taiwanensis* VLB120 CL3.3 on this substrate, they were cultivated in MSM containing glycerol as sole carbon source. When grown on 40 mM glycerol (Figure 6A), a final titer of 4.72 ± 0.01 mM 4-hydroxybenzoate was achieved, corresponding to a C-mol yield of 27.5%. A maximum production rate of 0.57 mmol ${\text{g}}_{\text{CDW}}^{-1}$ h^−1^ was reached after 16 h, which is a 1.8-fold increase compared to the same strains on glucose, and a 2.6-fold increase compared to the *P. putida* S12 strain of Verhoef et al. (2007) under similar conditions. The overexpression of *ppsA*-*pgi* in *P. taiwanensis* VLB120 CL3.3 further increased the titer to 5.1 ± 0.1 mM of 4-hydroxybenzoate with a C-mol yield of 29.6% (Figure 6B). Considering formation of *trans*-cinnamate as a byproduct of *Rt*PAL, *P. taiwanensis* VLB120 CL3 and *P. taiwanensis* VLB120 CL3.3 reached a total aromatics C-mol yield of 32.3% and 35.1%, respectively.

Increasing the initial glycerol concentration also increased product titers. This did, however, affect production efficiency, as C-mol yields of 23.9, 20.0, and 16.0% were reached using 80, 120, and 200 mM glycerol, respectively (Figure 6C). The relatively low yield achieved with 200 mM glycerol is likely, at least in part, caused by the fact that such a substrate concentration will lead to a nitrogen limitation under the conditions tested. Still, it is remarkable that 4-hydroxybenzoate production is more than twice as efficient from glycerol as from an equivalent concentration of glucose. For *P. putida* KT2440, it was shown that growth on glycerol leads to metabolic and regulatory rearrangements within the cell (Nikel et al., 2014a). Genes belonging to the Entner-Doudoroff pathway as well as stress-related genes are downregulated and enhanced expression of genes of the glyoxylate shunt as well as *pgi* was detected. In concert, these actions may result in increased availability of PEP and E4P which, together with the lower growth rate, could lead to a more efficient channeling of these central metabolites into the shikimate pathway.

Production of 4-hydroxybenzoate from glycerol was also investigated for strain *P. taiwanensis* VLB120 CL4 and *P. taiwanensis* VLB120 CL4.3. Unexpectedly, these strains only reached a final 4-hydroxybenzoate concentration of 0.44 ± 0.01 mM without and 0.43 ± 0.03 mM with *ppsA-pgi* overexpression, which is only 9.4% of the 4-hydroxybenzoate concentrations produced by *P. taiwanensis* VLB120 CL3. Additionally, for the strain *P. taiwanensis* VLB120 CL4, a residual concentration of 0.81 ± 0.01 mM tyrosine and 0.78 ± 0.01 mM for strain *P. taiwanensis* VLB120 CL4.3 was measured in the supernatant. These low titers may be caused by the lack of conversion of phenylalanine into *trans-*cinnamate, which could lead to an increased intracellular phenylalanine concentration, triggering feedback-inhibition mechanisms in the upstream pathway. This may be exacerbated by a reduced expression of *phhAB*, which is 20–30-fold less strongly induced on glycerol than on glucose in the 4-hydroxybenzoate producing *P. putida* S12palB1 (Verhoef et al., 2010). Indeed, the culture supernatant of the *Rs*TAL strains turned brownish during cultivation, hinting toward the accumulation of shikimate pathway intermediates, as described by Wynands et al. (2018).

Lignocellulosic biomass contains up to 25% pentose sugars such as xylose (Lee, 1997), making it a relevant alternative substrate especially on lignocellulosic hydrolysates. In contrast to e.g., *C. glutamicum* and *P. putida, P. taiwanensis* VLB120 is natively capable of assimilating xylose via the Weimberg pathway (Köhler et al., 2015). To exploit this trait for 4-hydroxybenzoate biosynthesis, production performance of strains *P. taiwanensis* VLB120 CL3 harboring *Rt*PAL and the *ppsA-pgi* overexpressing variant CL3.3, as well as their *Rs*TAL-harboring equivalents *P. taiwanensis* VLB120 CL 4 and CL4.3, was investigated in MSM containing 24 mM of xylose (Figure 6C). Strain CL3 produced 1.37 mM and strain CL3.3 (*ppsA-pgi* overexpression) 1.21 mM 4-hydroxybenzoate, which is 38% and 54%, respectively, less than on the equivalent amount of glucose. The counterparts CL4 and CL4.3. containing *Rs*TAL. reached similar titers of 1.0 mM and 1.1 mM. During its multi-step assimilation, xylose is converted to α-ketoglutarate and thus enters the primary metabolism at the level of the TCA cycle (Weimberg, 1961). This sets it apart from glucose and glycerol in that PEP and E4P as precursors for 4-hydroxybenzoate formation are formed by gluconeogenic reactions at a higher energetic expense compared to glycolysis. This is likely one explanation for the lower production performance. In accordance with Köhler et al. (2015), growth on xylose also resulted in decreased formation of biomass compared to glucose, with 0.83 g_CDW_ l^−1^ for strain CL3, 0.47 g_CDW_ l^−1^ for CL3.3, 0.8 g_CDW_ l^−1^ for CL4, and 0.58 g_CDW_ l^−1^ for strain CL4.3. In summary, the feasibility of 4-hydroxybenzoate production upon intrinsic use of xylose as sustainable carbon source could be demonstrated. However, the fact that neither the avoidance of *trans*-cinnamate formation through the use of *Rs*TAL, nor *ppsA-pgi* overexpression could considerably enhance 4-hydroxybenzoate titers, underlines that, as in the case of glucose and glycerol, production has to be tailored to the respective substrate. To this end, the insertion of a non-oxidative xylose metabolic pathway, likely coupled to adaptive laboratory evolution, may be a promising strategy (Meijnen et al., 2008, 2011).

### Production of 4-Hydroxybenzoate From Glycerol Via Pulsed Fed-Batch Cultivation

**Figure 7:**
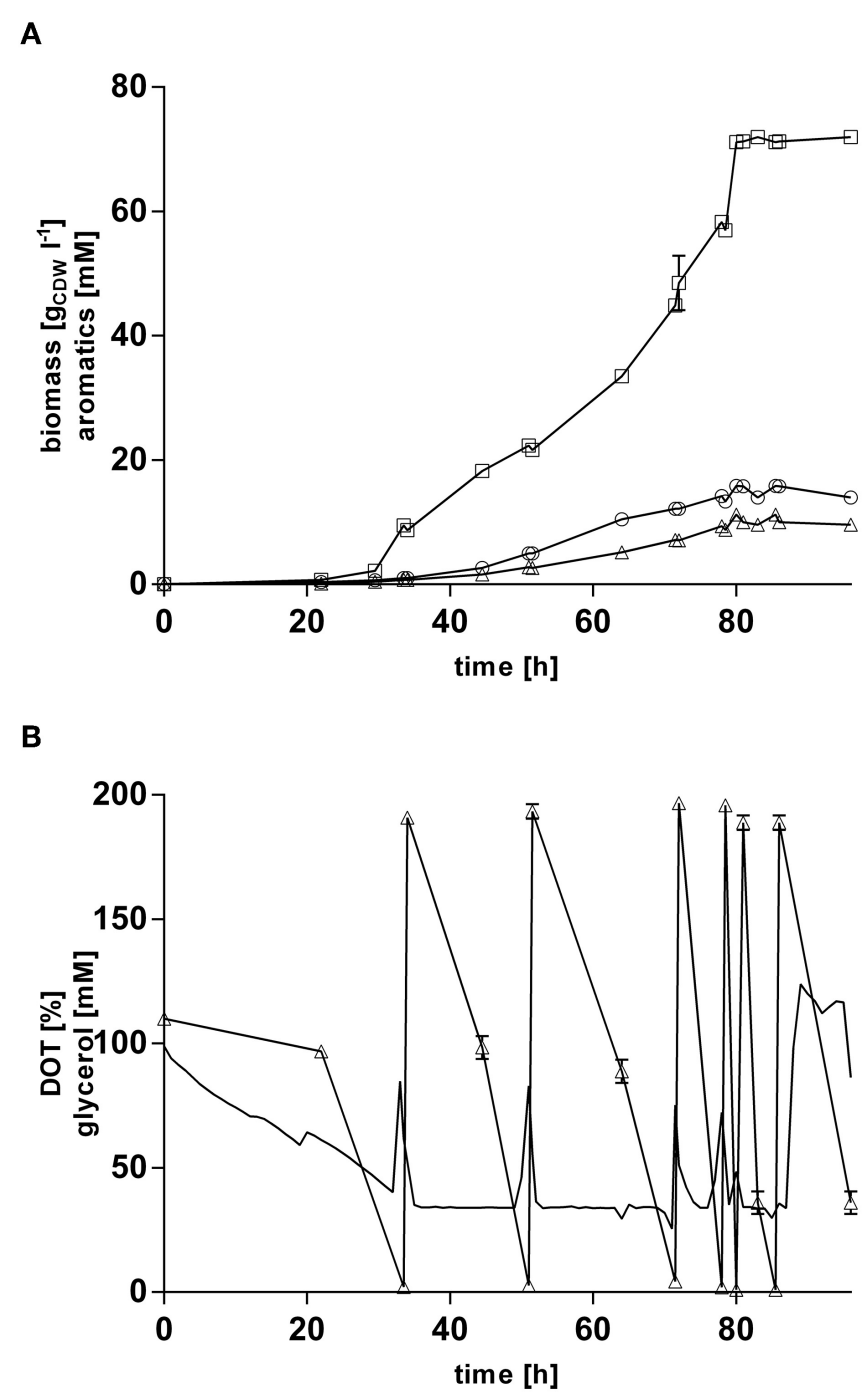
Glycerol pulsed fed-batch cultivation of *P. taiwanensis* VLB120 CL3.3. Cultivation took place in MSM containing an initial glycerol concentration of 120 mM as a sole carbon source. Expression of the ferulic operon and *ppsA-pgi* was induced by addition of 0.2 mM IPTG and 0.1 mM salicylate. Two biological replicates were cultivated. **(A)** Mean values of biomass (circles), 4-hydroxybenzoate (squares), *trans-*cinnamate (triangles) are shown. **(B)** Dissolved oxygen tension (no symbols) and glycerol (triangles). Dissolved oxygen tension values from one replicate are shown. Error bars indicate the deviation from the mean.

In order to increase final product titers beyond those enabled by simple shake-flask cultivations, strain *P. taiwanensis* VLB120 CL3.3 was cultivated in 1 l MSM with glycerol as sole carbon source in controlled bioreactors under pulsed fed-batch conditions (Figure 7). After an initial batch phase on 120 mM glycerol, the cultures were pulsed multiple times with 200 mM glycerol when an increase in the dissolved oxygen tension was observed, indicating a depletion of the carbon source. The reactors were titrated with ammonium hydroxide to maintain a neutral pH and to avoid a nitrogen limitation at higher cell densities. With this system, a final 4-hydroxybenzoate titer of 72.0 ± 0.96 mM (9.9 g l^−1^) was reached, and 11.2 ± 0.37 mM (1.7 g l^−1^) *trans*-cinnamate was produced, whereas no tyrosine was detected in the culture supernatant. After 85 h of cultivation, both growth and production ceased immediately, so that the last pulse of glycerol had no more effect on biomass and product formation and appeared to be only consumed for homeostasis. Excluding this final pulse, a 4-hydroxybenzoate C-mol yield of 19.2% and total aromatics C-mol yield of 23.0% were reached, with maximum production rate of 0.49 mmol ${\text{g}}_{\text{CDW}}^{-1}$ h ^−1^.

**Figure 8:**
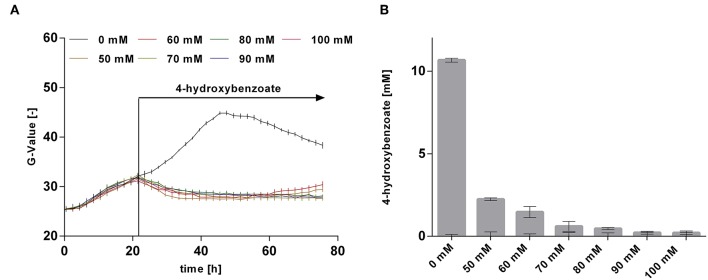
Growth and production upon a 4-hydroxybenzoate pulse. *P. taiwanensis* VLB120 CL3.3 was cultivated in 24-well System Duetz plates in a Growth Profiler in MSM containing 120 mM glycerol as sole carbon source. Each well was filled with a culture volume of 1,350 μl. At an OD_600_ of ~1, different concentrations of 4-hydroxybenzoate were applied as indicated, using 150 μl of a 10x stock solution for each concentration adjusted to pH 7, whereas water was added to the wells with no 4-hydroxybenzoate pulse. Growth **(A)** was monitored by image analysis and concentrations were measured immediately after addition and at the end of experiment. **(B)** Difference between 4-hydroxybenzoate concentrations just after the pulse and after 96 h. Values of triplicate samples are shown, error bars indicate standard error of the mean.

The rather abrupt cessation of growth and product formation is likely due to product toxicity, product inhibition, or both. In order to test this, batch cultures of *P. taiwanensis* VLB120 CL3.3 on MSM with 120 mM glycerol were pulsed with different concentrations of 4-hydroxybenzoate in the exponential growth phase after 23 h (Figure 8). At this point, the culture had already produced 3.7 ± 0.11 mM 4-hydroxybenzoate. Growth was monitored using a Growth Profiler® and 4-hydroxybenzoate concentrations were measured immediately after the pulse and at the end of the experiment. Compared to the control (no addition) which continued growing as expected and produced 14.4 ± 0.04 mM 4-hydroxybenzoate, a strong inhibition of growth was already observed at the lowest added concentration of 50 mM 4-hydroxybenzoate. Upon addition of 50 and 60 mM 4-hydroxybenzoate, a significant additional 2.2 ± 0.08 mM, respectively, 1.5 ± 0.33 mM 4-hydroxybenzoate were produced, which amounts to total titers of 5.9 ± 0.19 mM and 5.2 ± 0.33 mM, respectively. No significant increase in concentration was measured after a pulse of 70 mM 4-hydroxybenzoate and higher concentrations. These results are in good accordance with the final titer of 72 mM reached in the pulsed fed-batch cultivation, and indicate that both product toxicity and product inhibition are severe at this concentration of 4-hydroxybenzoate, and that product toxicity likely is the predominant factor. In comparison, an impressive tolerance of *C. glutamicum* toward up to 300 mM 4-hydroxybenzoate was shown by Kitade et al. (2018). During growth-arrested production, the engineered *C. glutamicum* strains produced 4-hydroxybenzoate with a remarkable titer of 37 g l^−1^ and a yield of 41% (mol/mol). This was achieved in a two-step-process comprising formation of biomass in a rich medium and subsequent high-cell density production in minimal medium. The *P. taiwanensis* VLB120 strains developed in this study, however, were subject to 4-hydroxybenzoate production using a fully mineral medium combined with cell growth, which hence was included in all yield calculations. The two-stage process reported by Kitade et al. (2018) could provide a promising approach to reduce product toxicity for *Pseudomonas*, given that the cells only need to survive, rather than grow. Alternatively, *in situ* product removal could be applied to circumvent the accumulation of inhibiting concentrations of 4-hydroxybenzoate, and thereby increase production performance of *P. taiwanensis* VLB120 hosts. Regarding this approach, strategies such as Calcium salt precipitation (Zambanini et al., 2016), co-crystallization (Urbanus et al., 2010) as well as reactive extraction (Schügerl and Hubbuch, 2005; Kreyenschulte et al., 2018) have been proven to be applicable for organic acids.

## Conclusion

This study describes the rational metabolic engineering of a *P. taiwanensis* VLB120 strain for high-yield microbial catalysis of glucose or glycerol into 4-hydroxybenzoate via the central metabolite L-tyrosine. This was achieved in a completely minimal medium, without the use of auxotrophies to force metabolic flux toward the product of interest. The formation of *trans-*cinnamate as a byproduct could be avoided through the use of the tyrosine-specific *Rs*TAL, but this only enabled efficient 4-hydroxybenzoate production from glucose, and not from glycerol. Large differences in product to substrate yields were achieved on these two carbon sources, with glycerol being the preferred substrate with C-mol yields up to 29.6% (= 0.19 g/g) as long as the less specific *Rt*PAL was used. To the best of our knowledge, this is the highest reported 4-hydroxybenzoate yield on a fully mineral medium. The unexpected interplay between the up- and downstream pathways of tyrosine should be further investigated, which would likely yield valuable insights into the underlying mechanisms of the fundamental synthetic biology concept of chassis and modules. Production of 4-hydroxybenzoate from xylose was also demonstrated, although in this case the relatively low performance compared to glucose or glycerol indicates that further improvement of central precursor supply is needed. In all, this work provides a significant advance in the efficiency of aromatics production in non-pathogenic Pseudomonads, the fundamentals of which can be applied to enable the sustainable production of 4-hydroxybenzoate. In addition, the development of the solvent-tolerant *P. taiwanensis* as aromatics platform can be further exploited for the biosynthesis of a wide range of other more toxic chemicals.

## Author Contributions

All authors saw and approved the manuscript. All authors contributed significantly to the work. NW conceived the project. NW, CL, and LB designed experiments and analyzed results. CL and NW wrote the manuscript with the help of LB, BW, and MO. CL performed experiments, supported by BW, MO, JB, and PM.

### Conflict of Interest Statement

NW is employed by the Institute for Bio-und Geosciences (IBG-1): Biotechnology, Forschungszentrum Jülich GmbH. The remaining authors declare that the research was conducted in the absence of any commercial or financial relationships that could be construed as a potential conflict of interest.
