# Supplementary material for: High-Yield Production of 4-Hydroxybenzoate From Glucose or Glycerol by an Engineered Pseudomonas taiwanensis VLB120
Source: Front Bioeng Biotechnol. 2019 Jun 12;7:130. doi: 10.3389/fbioe.2019.00130 (PMC6581684; doi:10.3389/fbioe.2019.00130)
Supplement: Supplementary file 1 [file Data_Sheet_1.docx]

Supplemental materials to:

High-yield production of 4-hydroxybenzoate from glucose or glycerol by an engineered *Pseudomonas taiwanensis* VLB120

Christoph Lenzen^1^, Benedikt Wynands^1,2^, Maike Otto^1,2^, Johanna Bolzenius^1^, Philip Mennicken^1^, Lars M. Blank^1^, Nick Wierckx^1,2*^

^1^RWTH Aachen University, Institute of Applied Microbiology iAMB, Worringerweg 1, 52074 Aachen, Germany.

^2^Forschungszentrum Jülich, Institute of Bio- and Geosciences IBG-1: Biotechnology, Wilhelm-Johnen-Straße, 52425 Jülich, Germany.

*** Correspondence:**Nick Wierckx
[n.wierckx@fz-juelich.de](mailto:n.wierckx@fz-juelich.de)

**Contents**

**Table S1: Oligonucleotides used in this study**

**Figure S1: Integration sites of the ferulic genes**

**Figure S2: Investigation of 4-hydroxybenzoate production in *P. taiwanensis* VLB120 CL3 deficient for catabolite repression regulators**

**Codon-optimized genes**

**Table S1: Oligonucleotides used in this study.** Restriction sites are underlined.

| **Primer** | **5’ – Sequence – 3’** | **Description** |
| --- | --- | --- |
| CL160 | GGCCGCGGCCGCGCGAATTCAGGAGGTACCAGCTATGAGCAAATATGAAGGCCGC | Cloning pBELK ferulic s:  Fwd *ech*- *vdh*-*fcs*, *EcoRI* |
| CL161 | CAGGTCGACTCTAGAGGATCCCGATGGCCGTGTCCTCGA | Cloning pBELK ferulic s:  Rev *ech*-*vdh*-*fcs*, BamHI |
| CL166 | CAATTTCACAGGTACCGAATTCAGGAGGTACCAGCTATGGCACCCTCGCTCGAC | Cloning pJT’ *Rt*PAL:  Fwd *Rt*PAL, *EcoRI* |
| CL167 | ACGTCGCATGCTCCTCTAGATTCTAAGCGAGCATCTTGAGGAG | Cloning pJT’ *Rt*PAL:  Rev *Rt*PAL, *XbaI* |
| CL353 | TAACAATTTCACAGGTACCGAATTCAGGAGGTGTGACCATGTTCATTGAAACCAACG | Cloning pJT’ *Tc*XAL:  Fwd *Tc*XAL, *EcoRI* |
| CL354 | GCCCGACGTCGCATGCTCCTCTAGATTAGAACATCTTGCCCAC | Cloning pJT’ *Tc*XAL:  Rev *Tc*XAL, *XbaI* |
| CL349 | TAACAATTTCACAGGTACCGAATTCAGGAGGTGTGACCATGAACACCATCAACGAG | Cloning pJT’ *Fj*TAL:  Fwd *Fj*TAL, *EcoRI* |
| CL350 | GCCCGACGTCGCATGCTCCTCTAGATTGTTAATCAGGTGGTCTTTC | Cloning pJT’ *Fj*TAL:  Rev *Fj*TAL, *XbaI* |
| CL444 | TAACAATTTCACAGGTACCGAATTCAGGAGGTACCAGCTATGAGCCCGCCGAAGCCC | Cloning pJT’ *Rs*TAL:  Fwd *Rs*TAL, *EcoRI* |
| CL445 | CACACCTCCTTCTAGATCACACCGGCGACTGCTG | Cloning pJT’ *Rs*TAL:  Rev *Rs*TAL, *XbaI* |
| CL213 | TCAAGATGCTCGCTTAGAATCTAGAAGGAGGTGTGACCATGAACTACCAAAACG | Cloning pJT’ *Rt*PALa:  Fwd *aroG^fbr^*, *XbaI* |
| CL214 | GCCCGACGTCGCATGCTCCTCTAGATTAGCCGCGCCGGGCCTT | Cloning pJT’ *Rt*PALa:  Rev *aroG^fbr^*, *XbaI* |
| CL195 | TCAAGATGCTCGCTTAGAATCTAGAAGGAGGTGTGACCATGAAC | Cloning pJT’ *Rt*PALat:  Fwd *aroG^fbr^-tyrA^fbr^*, *XbaI* |
| CL197 | GCCCGACGTCGCATGCTCCTCTAGATTACTGGCGGTTGTCGTTG | Cloning pJT’ *Rt*PALat:  Fwd *aroG^fbr^-tyrA^fbr^*, *XbaI* |
| CL196 | GCCCGACGTCGCATGCTCCTCTAGATTACAGCAGTTCTTTCGC | Cloning pJT’ *Rt*PALatt:  Fwd *aroG^fbr^-tyrA^fbr^-tktA*, *XbaI* |
| CL331 | TAGAAAACCTCCTTAGCATG | Cloning pBG14a/d/e/g/fg/ffg *Rt*PALat  Rev pBG14 backbone |
| CL332 | GAATTCGAGCTCGGTACC | Cloning pBG14a/d/e/g/fg/ffg *Rt*PALat  Fwd pBG14 backbone |
| CL333 | CATGCTAAGGAGGTTTTCTAATGGCACCCTCGCTCGAC | Cloning pBG14a/d/e/g/fg/ffg *Rt*PALat  Fwd *Rt*PAL-*aroG^fbr^-tyrA^fbr^* |
| CL334 | CGGGTACCGAGCTCGAATTCCTAAGCGAGCATCTTGAGGAGG | Cloning pBG14a/d/e/g/fg/ffg *Rt*PALat  Rev *Rt*PAL-*aroG^fbr^-tyrA^fbr^* |
| CL448 | TAGAAAACCTCCTTAGCATG | Cloning pBG14f *Rs*TAL at:  Rev pBG14f backbone |
| CL449 | GAATTCGAGCTCGGTACC | Cloning pBG14f *Rs*TAL at:  Fwd pBG14f backbone |
| CL450 | CATGCTAAGGAGGTTTTCTAATGAGCCCGCCGAAGCCC | Cloning pBG14f *Rs*TAL at:  Fwd *RsTAL* |
| CL445 | CACACCTCCTTCTAGATCACACCGGCGACTGCTG | Cloning pBG14f *Rs*TAL at:  Rev *RsTAL* |
| CL446 | GCCGGTGTGATCTAGAAGGAGGTGTGACCATGAAC | Cloning pBG14f *Rs*TAL at:  Fwd *aroG^fbr^-tyrA^fbr^* |
| CL447 | CGGGTACCGAGCTCGAATTCTTACTGGCGGTTGTCGTTGG | Cloning pBG14f *Rs*TAL at:  Rev *aroG^fbr^-tyrA^fbr^* |
| ARB6 | GGCACGCGTCGACTAGTACNNNNNNNNNNACGCC | Round 1 of arbitrary PCR |
| ARB2 | GGCACGCGTCGACTAGTAC | Round 2 of arbitrary PCR |
| pBAM-ME-I-Ext-R | CTCGTTTCACGCTGAATATGGCTC | Round 1 of arbitrary PCR for pBAMD1-2 |
| pBAM-ME-I-Ext-R | CAGTTTTATTGTTCATGATGATATA | Round 2 of arbitrary PCR for pBAMD1-2, sequencing |

**
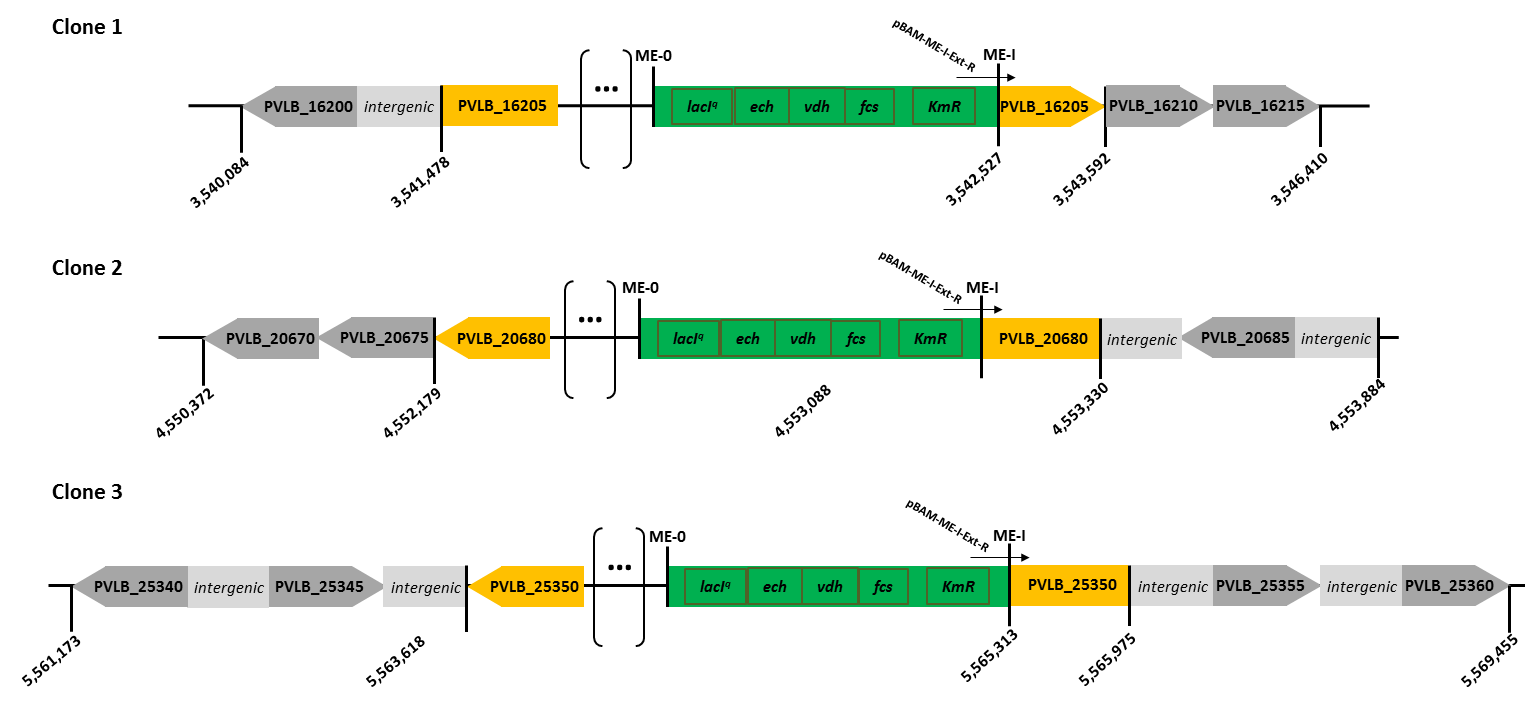
**

**Figure S1: Integration sites of the ferulic genes.** The operon was amplified from the genome of *P. putida* S12 and cloned into the pBELK mini-transposon vector. Tips of gene boxes indicate gene orientation, numbers indicate chromosomal location (bp). Genes are not drawn to scale. PVLB_16205, plug domain of the TonB-dependent receptor. PVLB_20680, SAM protein. PVLB_25350, sensory box protein. PVLB_16200, NAD(P)H-dependent glycerol-3-phosphate dehydrogenase. PVLB_16210, putative ABC transporter substrate-binding protein. PVLB_16215, integral membrane sensor hybrid histidine kinase. PVLB_20670, XRE family transcriptional regulator. PVLB_20675, type IV pilus biogenesis/stability protein PilW. PVLB_20685, multifunctional nucleoside diphosphate kinase. PVLB_25340, RipR family transcriptional regulator. PVLB_25345, glucose-6-phosphate 1-dehydrogenase. PVLB_23555, DNA-dependent helicase II. PVLB_25360, hypothetical protein. *lacI^q^, LacI* repressor gene and P_trc_ promoter*.*


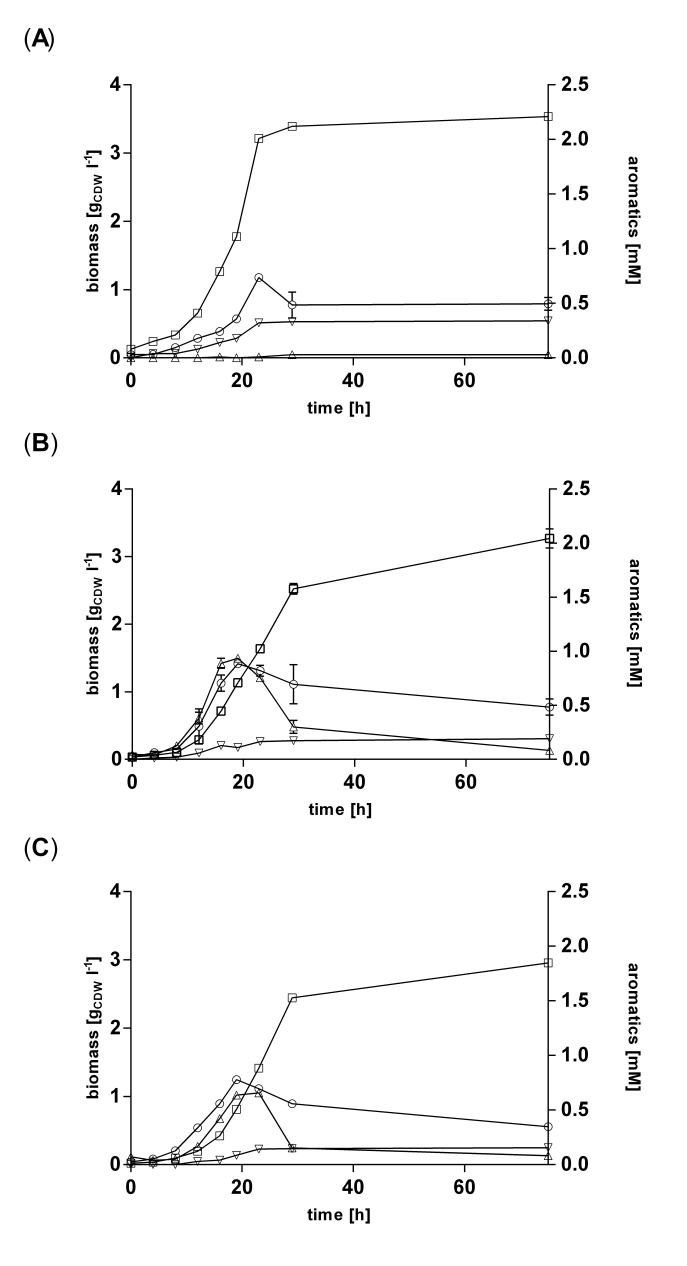


**Figure S2: Investigation of 4-hydroxybenzoate production in *P. taiwanensis* VLB120 CL3 deficient for catabolite repression regulators.** Cultivations of *P. taiwanensis* VLB120 CL3 (**A**), *P. taiwanensis* VLB120 CL3 Δ*crc* (**A**) and *P. taiwanensis* VLB120 CL3 Δ*hfq* (**C**) were carried out in MSM containing 20 mM glucose as a sole carbon source in 24-well System Duetz plates at 30 °C and 300 rpm. Expression of the ferulic operon was induced with 0.2 mM IPTG. Data of biomass (circles), 4-hydroxybenzoate (squares), tyrosine (triangles) and *trans-*cinnamate (inverted triangles) are shown. Error bars indicate standard errors of the mean.

**Codon-optimized genes**

*RsTAL*

5’-ATGTTGGCCATGAGCCCGCCGAAGCCCGCTGTGGAACTGGACCGTCACATCGACCTCGATGAAGCACACTCCGTGGCCAGCGGTGGCGCTCGCATCGTCCTGGCGCCGCCAGCACGCGATCGTTGCCGCGCCAGCGAAGCCCGTCTGGGCGCCGTGATTCGTGAAGCTCGTCATGTGTACGGCCTCACCACTGGGTTCGGTCCATTGGCCAATCGGCTCGTCTCCGGTGAAAATGTCCGTACTCTGCAAGCTAACCTGGTGCATCACCTGGCGTCGGGCGTAGGCCCGGTGCTCGATTGGACCACCGCGCGGGCAATGGTCTTGGCTCGCTTGGTGGCCATCGCCCAGGGCGCGTCGGGCGCCTCGGAGGGTACGATCGCGCGCCTCATCGACCTGCTGAACAGCGAACTCGCTCCCGCCGTGCCAATGCGGGGCACCGTCGGTGCCAGCGGCGACTTGACTCCGCTGGCTCACATGGTCCTGTGCCTCCAGGGCCGCGGGGATTTCCTCGACCGGGACGGCACCCGTCTGGACGGCGCGGAGGGCCTGCGCCGCGGCCGCCTGCAACCCTTGGACCTGTCGCACCGCGACGCCCTGGCGCTGGTCAACGGCACCAGTGCCATGACCGGCATCGCGCTGGTGAACGCTCACGCTTGCCGTCACCTGGGTAATTGGGCCGTGGCTCTGACCGCGCTGCTGGCGGAGTGTCTCGGGGGCCGCACCGAAGCGTGGGCCGCCGCACTCAGCGATCTGCGCCCTCACCCCGGCCAAAAGGACGCTGCTGCTCGCCTGCGCGCGCGGGTGGACGGTAGCGCCCGCGTCGTCCGCCACGTGATTGCCGAACGCCGCCTGGGTGCGAGCGATATCGGGACTGAACCAGAAGCGGGGCAGGACGCGTACAGCCTCCGCTGCGCACCGCAGGTACTGGGCGCGGGCTTCGACACGTTGGCTTGGCATGACCGGGTCCTGACCATCGAGCTGAACGCAGTCACTGACAACCCGGTCTTCCCGCCGGACGGCAGCGTGCCGGCGCTGCACGGGGGTAACTTTATGGGCCAGCACGTCGCGCTGACCAGTGACGCACTGGCCACCGCCGTCACCGTCCTGGCCGGTCTGGCGGAGCGGCAGATCGCGCGGCTGACTGACGAACGCCTGAACCGTGGCTTGCCGCCGTTCCTGCACCGCGGCCCCGCCGGTCTCAACTCCGGCTTCATGGGCGCCCAGGTCACCGCCACCGCTCTGCTGGCAGAAATGCGCGCCACCGGCCCCGCGAGCATTCACAGCATCAGCACCAACGCCGCAAACCAGGACGTCGTCTCCCTGGGCACCATCGCAGCCCGTTTGTGCCGTGAAAAGATCGACCGCTGGGCAGAAATCCTCGCCATCCTGGCCCTGTGCCTGGCCCAGGCGGCCGAACTGCGGTGCGGCAGCGGCCTCGACGGCGTGTCGCCAGCCGGCAAGAAATTGGTCCAGGCCTTGCGCGAACAGTTCCCTCCGCTGGAGACCGATCGCCCCCTGGGGCAAGAAATCGCTGCGTTGGCCACCCACCTCTTGCAGCAGTCGCCGGTGTGA – 3’

*FjTAL*

5’-ATGAACACCATCAACGAGTATCTGTCGTTGGAGGAGTTCGAGGCCATTATTTTCGGGAACCAAAAGGTCACCATCAGCGACGTCGTCGTCAATCGCGTCAACGAATCCTTCAACTTTCTGAAGGAGTTCAGCGGCACAAGGTGATCTACGGCGTGAACACCGGCTTTGGCCCGATGGCGCAGTACCGTATCAAGGAGAGCGATCAAATCCAGCTGCAATACAACCTGATCCGCAGCCATAGCTCGGGGACCGGGAAGCCGCTGTCCCCGGTCTGTGCCAAGGCCGCTATCTTGGCCCGCCTGAACACCCTGAGCCTCGGGAACAGCGGCGTGCACCCTAGCGTAATTAATTTGATGAGCGAACTGATCAACAAGGACATCACGCCGCTGATCTTCGAACACGGCGGGGTGGGCGCGAGTGGCGACCTGGTACAGCTGTCGCACTTGGCGCTGGTCCTGATCGGCGAAGGTGAAGTCTTCTACAAAGGCGAGCGCCGTCCGACCCCGGAGGTGTTTGAAATCGAGGGCCTCAAGCCAATCCAGGTCGAGATCCGCGAAGGCCTGGCCTTGATCAACGGTACGAGCGTGATGACCGGCATCGGTGTCGTGAACGTATATCATGCGAAAAAGTTGCTGGACTGGTCGCTGAAGTCCAGCTGCGCCATCAACGAACTGGTCCAAGCCTACGACGATCACTTCAGCGCCGAACTGAACCAGACGAAACGCCACAAAGGCCAACAGGAGATCGCGCTGAAGATGCGTCAGAACCTCAGCGATTCCACCTTGATCCGGAAGCGCGAGGACCACCTGTACTCCGGCGAAAACACCGAGGAAATCTTCAAGGAAAAAGTTCAGGAGTACTACAGCCTGCGCTGTGTGCCGCAAATCCTGGGTCCTGTGCTGGAGACGATCAACAACGTTGCCTCGATCCTCGAAGATGAGTTTAACAGCGCGAATGATAACCCGATTATCGACGTCAAGAACCAACACGTCTACCACGGCGGCAACTTCCACGGCGACTACATTTCCCTGGAAATGGACAAACTGAAGATCGTGATCACCAAGCTGACGATGCTGGCCGAACGCCAACTGAATTACTTGCTGAACAGCAAGATCAATGAGCTGCTGCCCCCTTTTGTCAACCTGGGTACGCTGGGTTTCAACTTCGGTATGCAAGGGGTGCAATTTACCGCCACCAGCACCACCGCGGAATCGCAGATGCTGAGTAACCCGATGTACGTTCACTCCATTCCGAACAACAACGACAATCAGGACATTGTGAGCATGGGCACGAACTCCGCGGTAATCACCTCCAAAGTGATCGAAAACGCCTTCGAGGTACTGGCCATTGAAATGATCACCATTGTTCAGGCGATTGACTACCTGGGTCAGAAGGACAAGATCAGCTCGGTGTCGAAGAAGTGGTACGATGAAATCCGCAACATCATCCCTACGTTCAAAGAAGACCAAGTTATGTATCCTTTCGTGCAGAAGGTGAAAGACCACCTGATTAACAACTGA – 3’

*TcXAL*

5’- ATCGCGGGGGCCATCTGCGGCCACCCGGATGTCAAGGTATTCGACACCGCCGCCAGCCCACCTACCGTGCTGACCAGCCCCGAGGCAATCGCCAAATACGGCCTGAAAACCGTCAAGCTGGCCAGCAAGGAGGGCCTGGGTTTGGTGAACGGCACTGCCGTCTCGGCAGCGGCGGGCGCCTTGGCGCTGTACGATGCCGAATGCCTGGCCATCATGAGCCAGACCAACACCGTCCTGACCGTCGAAGCCCTGGACGGTCACGTAGGTAGCTTCGCGCCGTTCATCCAAGAAATCCGTCCACACGCCGGCCAAATCGAAGCTGCGCGCAACATCCGCCATATGCTCGGCGGCAGCAAATTGGCAGTCCACGAGGAATCGGAGCTGTTGGCCGACCAAGATGCAGGCATCCTGCGCCAGGACCGCTACGCTCTGCGTACCAGCGCCCAATGGATCGGGCCGCAGCTGGAGGCCCTGGGCCTGGCCCGTCAGCAGATCGAGACTGAGCTGAACTCCACCACCGATAACCCACTGATTGACGTGGAGGGGGGCATGTTCCACCATGGCGGGAACTTCCAGGCCATGGCTGTCACGTCCGCTATGGACTCCGCCCGCATCGTACTGCAGAACTTGGGCAAACTGTCCTTCGCCCAAGTAACGGAGCTGATCAACTGCGAAATGAACCATGGCCTGCCATCCAACTTGGCTGGTTCGGAACCGTCCACCAACTATCACTGCAAGGGCCTGGATATCCACTGTGGGGCGTACTGCGCCGAGCTGGGCTTCTTGGCGAACCCTATGTCGAACCATGTCCAGAGCACCGAGATGCATAACCAGAGCGTGAACTCGATGGCCTTTGCCAGCGCCCGCCGCACGATGGAAGCAAACGAAGTATTGAGCCTGCTGCTGGGCAGCCAGATGTACTGTGCCACCCAGGCCCTGGACCTGCGCGTGATGGAAGTGAAGTTCAAGATGGCAATTGTCAAACTCCTCAACGAGACGCTCACGAAGCACTTCGCCGCGTTCCTGACCCCAGAACAACTCGCCAAGCTCAATACTCATGCCGCGATCACCCTGTATAAGCGCCTGAACCAGACCCCAAGCTGGGACTCGGCGCCGCGCTTCGAAGACGCCGCCAAGCACCTGGTGGGCGTCATCATGGATGCGCTGATGGTGAACGATGATATCACCGACCTGACCAACCTCCCAAATGGAAGAAGGAGTTCGCTAAAGAGGCGGGCAACTTGTATCGCTCGATCCTCGTAGCCACCACCGCAGACGGTCGCAACGACCTGGAGCCGGCTGAGTACCTGGGCCAGACCCGCGCTGTGTACGAGGCGGTCCGGTCCGAATTGGGCGTGAAGGTCCGCCGCGGCGACGTGGCGGAGGGCAAGAGCGGTAAATCGATCGGCAGCAGCGTCGCCAAAATCGTCGAGGCGATGCGGGACGGTCGCCTGATGGGCGCGGTGGGCAAGATGTTCTGA- 3’

*aroG*^fbr^

5’ -

ATGAACTACCAAAACGATGATCTGCGCATCAAGGAAATCAAAGAACTCCTGCCTCCCGTAGCACTCCTGGAGAAGTTCCCAGCGACCGAGAACGCCGCCAATACCGTGGCCCACGCCCGTAAGGCCATCCACAAGATCCTCAAAGGCAACGACGATCGCTTGTTGGTCGTCATCGGGCCGTGCTCGATCCACGACCCGGTCGCGGCCAAGGAGTACGCGACCCGCCTGCTGGCGCTCCGCGAAGAGCTGAAAGACGAACTGGAAATCGTCATGCGCGTGTATTTCGAAAAGCCACGGACTACTGTGGGCTGGAAGGGTTTGATCAACGATCCTCATATGGACAACAGCTTCCAGATTAACGACGGTCTCCGCATCGCCCGCAAATTGCTGCTCGATATCAACGACAGCGGCCTGCCAGCAGCCGGCGAGTTCCTCAATATGATCACCCCCCAGTATCTCGCAGACCTGATGAGCTGGGGCGCTATTGGCGCGCGGACCACCGAGTCGCAAGTACACCGGGAACTGGCTTCGGGCCTGAGC

TGTCCTGTCGGCTTCAAGAACGGGACCGACGGCACCATCAAGGTGGCGATCGACGCCATCAACGCGGCGGGCGCCCCACACTGCTTCTTGAGTGTCACCAAGTGGGGCCACAGTGCGATTGTCAATACCTCCGGCAACGGGGACTGCCACATTATCCTGCGGGGCGGCAAGGAACCCAACTACAGCGCGAAGCACGTGGCCGAGGTTAAAGAGGGCCTCAACAAGGCTGGGCTGCCGGCCCAGGTGATGATCGACTTCAGCCATGCGAATAGCTCCAAGCAGTTCAAGAAACAGATGGATGTTTGTGCCGATGTGTGTCAGCAGATCGCCGGTGGCGAAAAAGCCATCATCGGCGTTATGGTGGAGAGCCACCTCGTCGAGGGCAACCAGTCGCTGGAAAGCGGCGAGCCTCTGGCTTACGGTAAAAGCATCACCGACGCCTGCATCGGCTGGGAGGACACCGACGCCCTGCTGCGCCAGCTGGCGAATGCCGTGAAGGCCCGGCGCGGCTAA – 3`

*tyrA*^fbr^

5`-

ATGGTTGCAGAACTGACCGCGCTGCGTGACCAAATCGACGAGGTGGACAAAGCACTGTTGAACCTCCTGGCTAAGCGCCTGGAGCTGGTGGCCGAGGTGGGCGAGGTCAAAAGTCGCTTTGGCCTGCCTATTTACGTTCCTGAGCGCGAGGCAAGCATCCTGGCAAGCCGTCGCGCCGAGGCTGAGGCCCTGGGTGTGCCCCCCGACTTGATCGAGGATGTCCTGCGCCGCGTGATGCGTGAAAGCTACAGCTCGGAGAACGACAAGGGCTTCAAGACCTTGTGCCCCTCCTTGCGCCCAGTCGTGATTGTCGGCGGGGGGGGCCAGATGGGCCGTTTGTTCGAAAAGATGCTCACGCTGAGCGGCTACCAGGTGCGCATCCTGGAACAGCACGACTGGGACCGGGCGGCCGACATCGTCGCTGATGCCGGTATGGTAATCGTAAGCGTCCCCATCCATGTGACCGAACAGGTGATTGGCAAGCTGCCTCCGTTGCCGAAGGACTGCATCCTGGTGGATCTCGCGAGCGTGAAGAACGGCCCGCTGCAAGCGATGCTGGTTGCCCATGATGGCCCGGTGCTGGGCCTGCACCCAATGTTCGGCCCAGACAGCGGCTCGCTGGCAAAGCAAGTCGTGGTGTGGTGCGACGGTCGCAAACCCGAGGCGTATCAATGGTTCTTGGAACAGATCCAGGTTTGGGGTGCCCGTTTGCACCGCATCTCCGCAGTCGAGCATGACCAGAATATGGCTTTCATCCAGGCCCTGCGCCATTTCGCCACCTTCGCGTACGGCCTGCACCTGGCAGAGGAGAACGTGCAGCTGGAGCAGCTGCTGGCACTCAGCTCCCCCATCTACCGCCTGGAGCTGGCCATGGTCGGTCGCCTGTTCGCCCAGGACCCGCAGCTGTACGCGGACATCATCATGTCGTCCGAACGCAATCTCGCGCTGATCAAGCGCTATTATAAACGCTTCGGCGAGGCGATCGAACTGCTGGAACAGGGCGATAAGCAAGCCTTCATCGACTCGTTCCGCAAGGTGGAACATTGGTTCGGCGACTATGTCCAGCGCTTCCAGTCGGAGTCGCGCGTTCTGTTGCGTCAAGCCAACGACAACCGCCAGTAA – 3`
